# Root‐specific expression of chickpea cytokinin oxidase/dehydrogenase 6 leads to enhanced root growth, drought tolerance and yield without compromising nodulation

**DOI:** 10.1111/pbi.13378

**Published:** 2020-09-01

**Authors:** Hitaishi Khandal, Santosh Kumar Gupta, Vikas Dwivedi, Drishti Mandal, Nilesh Kumar Sharma, Niraj Kumar Vishwakarma, Lalita Pal, Megha Choudhary, Aleena Francis, Paheli Malakar, Nagendra Pratap Singh, Kapil Sharma, Senjuti Sinharoy, Narendra Pratap Singh, Rameshwar Sharma, Debasis Chattopadhyay

**Affiliations:** ^1^ National Institute of Plant Genome Research New Delhi India; ^2^ Repository of Tomato Genomics Resources Department of Plant Sciences University of Hyderabad Hyderabad India; ^3^ Indian Institute of Pulses Research Kanpur India

**Keywords:** *CaCKX6*, *Cicer arietinum* L., cytokinin, drought tolerance, mineral, root

## Abstract

Cytokinin group of phytohormones regulate root elongation and branching during post‐embryonic development. Cytokinin‐degrading enzymes cytokinin oxidases/dehydrogenases (CKXs) have been deployed to investigate biological activities of cytokinin and to engineer root growth. We expressed chickpea cytokinin oxidase 6 (*CaCKX6*) under the control of a chickpea root‐specific promoter of *CaWRKY31* in *Arabidopsis thaliana* and chickpea having determinate and indeterminate growth patterns, respectively, to study the effect of cytokinin depletion on root growth and drought tolerance. Root‐specific expression of *CaCKX6* led to a significant increase in lateral root number and root biomass in *Arabidopsis* and chickpea without any penalty to vegetative and reproductive growth of shoot. Transgenic chickpea lines showed increased *CKX* activity in root. Soil‐grown advanced chickpea transgenic lines exhibited higher root‐to‐shoot biomass ratio and enhanced long‐term drought tolerance. These chickpea lines were not compromised in root nodulation and nitrogen fixation. The seed yield in some lines was up to 25% higher with no penalty in protein content. Transgenic chickpea seeds possessed higher levels of zinc, iron, potassium and copper. Our results demonstrated the potential of cytokinin level manipulation in increasing lateral root number and root biomass for agronomic trait improvement in an edible legume crop with indeterminate growth habit.

## Introduction

Chickpea (*Cicer arietinum* L.), a grain legume with indeterminate growth habit (Hegde, 2011), is the second most economically important pulse crop, having an annual production of more than 14.2 million tons (FAOSTAT, 2016) with most of its cultivation in the developing countries. It is the major protein source for the vegetarian population in the growing countries. Additionally, legumes develop a symbiotic relationship with rhizobia and form root nodules allowing them to acquire fixed nitrogen. It is integral to the cereal‐based intercropping system as it improves soil fertility. Spatio‐temporal regulation of cytokinin is very important during nodule development. Therefore, it is important to investigate whether manipulation of root cytokinin level can be applied to legume or not. Chickpea is generally grown in the marginal lands with residual moisture and, therefore, yields far below than its potential (FAOSTAT, 2009). Chickpea yield is severely affected by terminal drought (Kashiwagi *et al.*, 2006). Several efforts are being made for biofortification of chickpea with zinc and iron (Tan *et al.*, 2018; Upadhyaya *et al.*, 2016). Low genetic base in chickpea landraces due to domestication bottlenecks impedes its improvement by conventional breeding (Abbo *et al.*, 2000; Abbo and Rubin, 2000).

Root traits remain a less‐explored area for crop improvement (Herder *et al.*, 2010; Wachsman *et al.*, 2015). Cultivars with a larger root system are desirable for obtaining plants with improved growth and yield characters and abiotic stress tolerance (Fang *et al* *.*, 2017). A direct correlation between the root size and resistance to water deficit has been shown in many crop plants. Plants with larger root systems displayed an increased ability to compete for nutrients and to survive under conditions of nutrient deficiency (Brown *et al.*, 1989; Henry, 2012; Kondo *et al.*, 1999; Krishnamurthy *et al.*, 1996; Morita and Nemoto, 1995; Passioura, 1982; Saxena *et al.*, 1994; Steele *et al.*, 2006). The ability of the root system to adjust in response to various abiotic stresses is an important aspect of the plant’s performance (Smith and De Smet, 2012). Root biomass and availability of soil resources including water and minerals have a strong impact on seed yield (Mandal *et al.*, 2010). For instance, chickpea cultivars with higher root length density are more drought‐tolerant and high yielding under terminal drought (Kashiwagi *et al.*, 2006; Singh *et al.*, 2016).

The root system is highly plastic in its development and adaptation to variable environmental conditions such as uneven water and nutrient distribution. This plasticity is essential for the acquisition of water and nutrient in a heterogeneous environment. Vertical root growth and root branching, that is production of lateral roots (LRs), constitute root system architecture (RSA) of the plant. Various phytohormones, most importantly auxin and cytokinin, play a vital role in root growth and development. In younger root tissues, cytokinin promotes cell differentiation (Werner *et al.*, 2001; Werner *et al.*, 2003; Dello Ioio *et al.*, 2008). The mechanism of initiation and elongation of LR is well studied in *Arabidopsis*. Local activation of auxin signalling precedes LR primordia formation (De Smet *et al.*, 2007; Möller *et al.*, 2017; Van Norman *et al.*, 2013). On the contrary, cytokinin treatment inhibits LR formation (Böttger, 1974; Goodwin and Morris, 1979; Wightman *et al.*, 1980). *Arabidopsis* mutants defective in cytokinin response displayed increased root branching (Mason *et al.*, 2005; Riefler *et al.*, 2006; To and Kieber, 2008).

Active cytokinin pool in the plant is regulated by reversible glycosylation, conversion to cytokinin nucleotides by adenine phosphoribosyltransferase genes, and through the irreversible breakdown of cytokinin by cytokinin oxidase/dehydrogenases (*CKX*s) (Sakakibara, 2006). *Arabidopsis* genome encodes seven *CKX* genes, and some of them were deployed to study the role of cytokinin in plant development. Overexpression of *AtCKX1* resulted in LR primordia formation in close proximity in *Arabidopsis* due to cell division activity in several pericycle cells (PCs) in a stretch. Seven *AtCKX* genes displayed different tissue‐specific expression and subcellular localization (Schmülling, 2002; Werner *et al.*, 2003; Werner *et al.*, 2001). *AtCKX* genes express during LR formation (Chang *et al.*, 2015). Grafting of *AtCKX1*‐overexpressing tobacco shoot on wild‐type root and *vice‐versa* did not alter the phenotypes of roots and shoots derived from the other plants indicating that the effect of cytokinin degradation is limited within the *CKX1*‐expressing tissue only (Werner *et al.*, 2010). *AtCKX3* and *AtCKX5* were reported to regulate reproductive meristem activity in *Arabidopsis* (Bartrina *et al.*, 2011). Expression of *AtCKX1* and *AtCKX3* under predominantly root‐expressing promoters of *AtWRKY6* or *AtPYK10* caused increased root growth without compromising shoot growth (Werner *et al.*, 2010). Tobacco plants expressing *AtCKX1 and AtCKX2* in root showed improved drought tolerance (Li *et al.*, 2017; Macková *et al.*, 2013). The strategy of manipulating root cytokinin by using *Arabidopsis CKX1 & CKX2* has been applied to barley, poplar and oilseed rape, respectively, for agronomic trait improvement (Nehnevajova *et al.*, 2019; Li *et al.,* 2019b; Ramireddy *et al.*, 2018a). This has led to enhanced root growth and drought tolerance. However, information about *CKX* gene of an edible legume crops and their application to improve agronomic traits is lacking.

There is a serious need to develop high‐yielding chickpea varieties that can tolerate periodic water‐limited conditions and possess higher seed mineral contents. In this study, chickpea *CKX6* gene (*CaCKX6*) has been used to modulate cytokinin level in a targeted tissue‐specific manner in root using chickpea *WRKY31* (*CaWRKY31*) promoter to investigate the effect of cytokinin depletion in root growth and architecture and subsequently, on yield and productivity of chickpea.

## Results

### Chickpea genome encodes ten cytokinin oxidase/dehydrogenase genes

Overexpression of *AtCKX1‐6* in *Arabidopsis* under the constitutive *35S CaMV* promoter was reported to yield similar qualitative phenotypes of enhanced root growth with retarded shoot growth in the transgenic lines overexpressing individual *CKX* genes (Werner *et al.*, 2003). However, quantitative phenotypic analyses of only *AtCKX1‐4* overexpressing lines were presented in that report. *AtCKX7* overexpression resulted in retarded root growth in *Arabidopsis* (Kӧllmer *et al.*, 2014). Detailed phenotypic analysis of plants with overexpression or root‐specific expression of *AtCKX6* has not been reported yet. Functional analysis of *CKX6* gene in legume crops has not been reported so far. A search in the annotated chickpea genome and transcriptome sequences available in public databases (Garg *et al.*, 2011; Varshney *et al.*, 2013) showed the presence of ten non‐redundant genes that encode proteins having sequence similarities with seven *Arabidopsis* CKX proteins (Schmülling *et al.*, 2003). They were annotated as *C. arietinum* CKX (*CaCKX*). Genes encoding two chickpea CaCKX2‐like proteins (XP_004488073.1 and XP_004488074.1) are tandemly repeated in the chickpea genome assembly and most probably, one of those originated due to local gene duplication ~5.7 million years ago as calculated using the rate of synonymous substitution (K_s_ 0.07). Similar search resulted in the identification of nine *CKX*‐encoding genes in the annotated *Medicago truncatula* genome assembly (Young *et al.*, 2011). Amino acid (aa) sequences of CKX proteins from both the legume species and those of *Arabidopsis* were aligned to construct a phylogenetic tree (Figure S1a). The tree placed two Ca*CKX6*‐like proteins (XP_004514814.1, XP_012574863.1) and two *MtCKX6*‐like proteins (XP_003599606.1, XP_003605865.1) in the same clade with *AtCKX6* in close proximity (Figure S1b). The clade of CKX1 proteins of all three species was close to *CKX6* clades. Two *CKX6*‐like proteins, one each from *Medicago* and chickpea (XP_003605865.1 and XP_012574863.1, respectively), showed size and sequence similarity to *AtCKX6*. The other two *CKX6*‐like proteins of these two legume species (XP_00451481.1 of chickpea and XP_003599606.1 of *Medicago*) showed the presence of extra twenty amino acids at their N‐terminus. These CKX proteins are longer by 21 and 14 aa, respectively, in comparison with AtCKX6 and longer than all the CKX proteins of *Arabidopsis* except AtCKX1. In order to characterize an unusually long CaCKX6, XP_00451481.1 of chickpea was selected for this study and is referred to as CaCKX6 onwards. The *CaCKX6* gene is 4935 bp long with five exons. The protein‐coding sequence (CDS) is of 1665 bp encoding a 554 aa long peptide (Figure S1c). At present, the chickpea genome assembly scaffold that encodes this gene has not been anchored to any chromosome. The peptide possesses predicted FAD‐ and cytokinin‐binding domains in 97–239 aa and 271–547 aa regions, respectively, characteristics of a cytokinin dehydrogenase enzyme.

### Subcellular localization and fractionation of CaCKX6 protein

**Figure 1:**
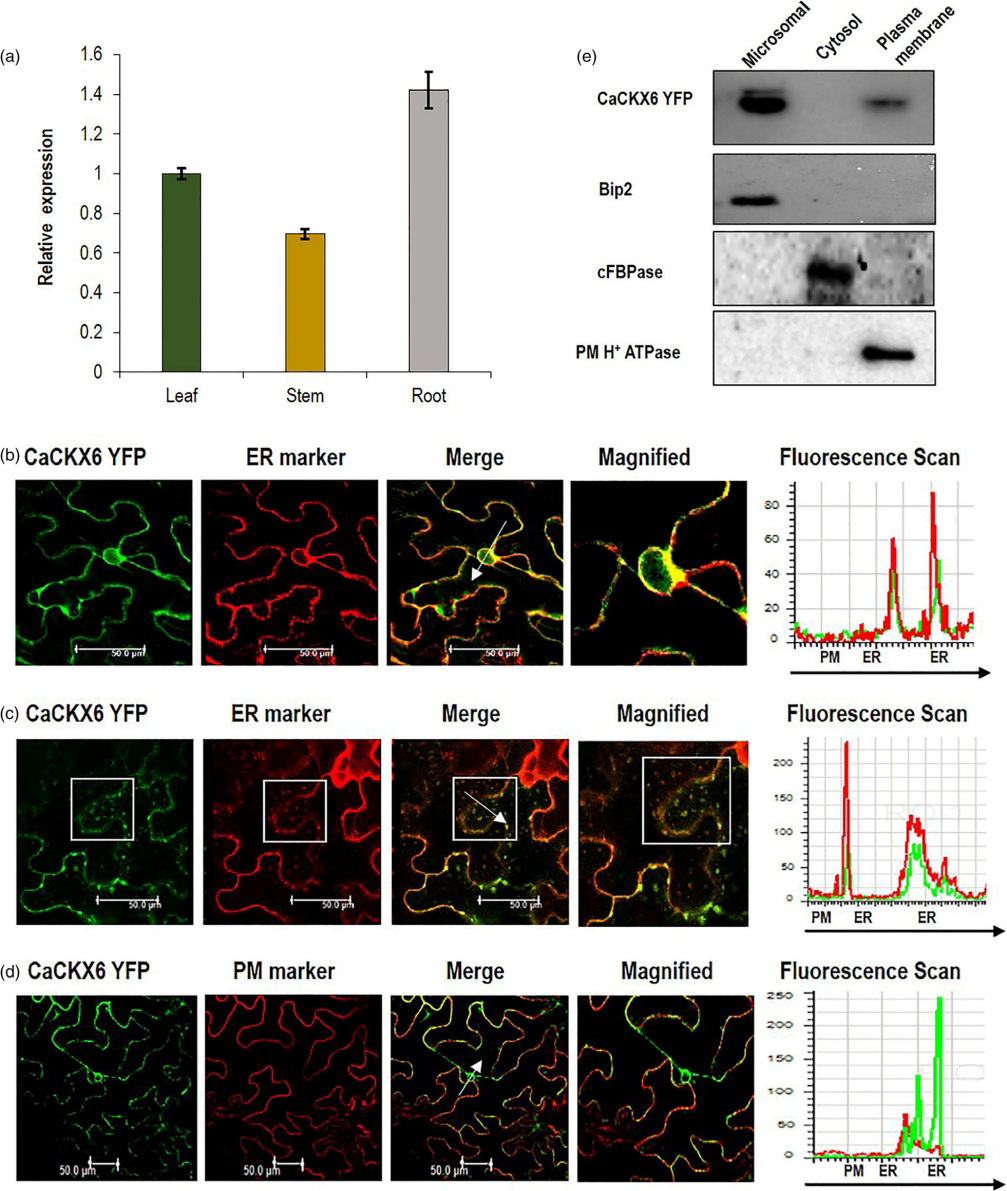
Relative expression, subcellular localization and fractionation of CaCKX6 (a) Expression of *CaCKX6* gene in root, stem and leaf of chickpea (var. Pusa‐362) relative to the expression level in leaf. RT‐qPCR was performed using three biological replicates and *CaEF‐1α* (NM_001365163.1) as internal control. (b–d) Subcellular localization of Ca*CKX6*. CaCKX6‐YFP along with endoplasmic reticulum (ER) marker (CD3‐959 mCherry) or plasma membrane (PM) marker (CD3‐1007 mCherry) was co‐agroinfiltrated in *N. benthamiana* leaves and was visualized by confocal microscopy after 2–3 days. Merged image indicates co‐localization of Ca*CKX6* and the marker protein. The region and direction of fluorescence intensity measurements are shown by white arrow. (e) Cellular extracts from CaCKX6‐YFP‐infiltrated leaves of *N. benthamiana* were fractioned into microsomal, cytosol and plasma membrane fractions. Immunoblot of all the fractions was probed with anti‐GFP, anti‐Bip2 (ER marker), anti‐cFBPase (cytosol marker) and anti‐PM H^+^ ATPase (plasma membrane marker) antibodies.

*CaCKX6* gene expresses the most in the root, more than twofold higher than that in the stem, while, expression in leaf was almost 1.5‐fold of that in stem (Figure 1a). To explore subcellular localization of CaCKX6 protein, the *CaCKX6*‐*YFP* construct was agroinfiltrated in *Nicotiana benthamiana* leaves along with endoplasmic reticulum (ER) marker (CD3‐959 mCherry) and a plasma membrane (PM) marker (CD3‐1007 mCherry) (Nelson *et al.*, 2007). The YFP distribution was monitored in the abaxial epidermal leaf cells using confocal microscopy. Fluorescence overlay analysis and fluorescence intensity scans (white arrow mark) of the observed green (YFP) and red (mCherry) fluorescence patterns suggest localization of CaCKX6 in ER. Outer nuclear membrane is known to be contiguous with the endoplasmic reticulum. Presence of punctated structure in cytoplasm and YFP labelled ring‐like fluorescence of nuclear envelope around the non‐fluorescent nucleoplasm, co‐localized with ER marker which further suggests that CaCKX6 is localized in ER (Figure 1b and c). A minor co‐localization of CaCKX6 with plasma membrane marker was also observed. Both of these co‐localizations (ER and PM) were verified by fluorescence intensity scans and subcellular fractionation followed by Western blot (Figure 1d and e).

### Overexpression of *CaCKX6* in *Arabidopsis* resulted in robust root and stunted shoot

Overexpression of *CaCKX6* under constitutive 35S‐CaMV promoter (*35S::CKX6*) in *Arabidopsis* resulted in more than 1.3‐fold increase in primary root length and more than twofold lateral root number when vertically grown in MS media for ten days. The transgenic lines showed better soil‐holding capacity when grown in pots for ten days after germination. However, shoots of the pot‐grown plants were severely stunted with smaller leaves and delayed reproductive maturity, as observed before in case of *AtCKX1* overexpression (Werner *et al.*, 2003). Inflorescence height and seed yield were less in these transgenic lines than that of wild‐type (WT) plants (Figure S2a–h).

### Root‐specific expression of *CaCKX6* in *Arabidopsis* and chickpea resulted in increased root biomass without any yield penalty

**Figure 2:**
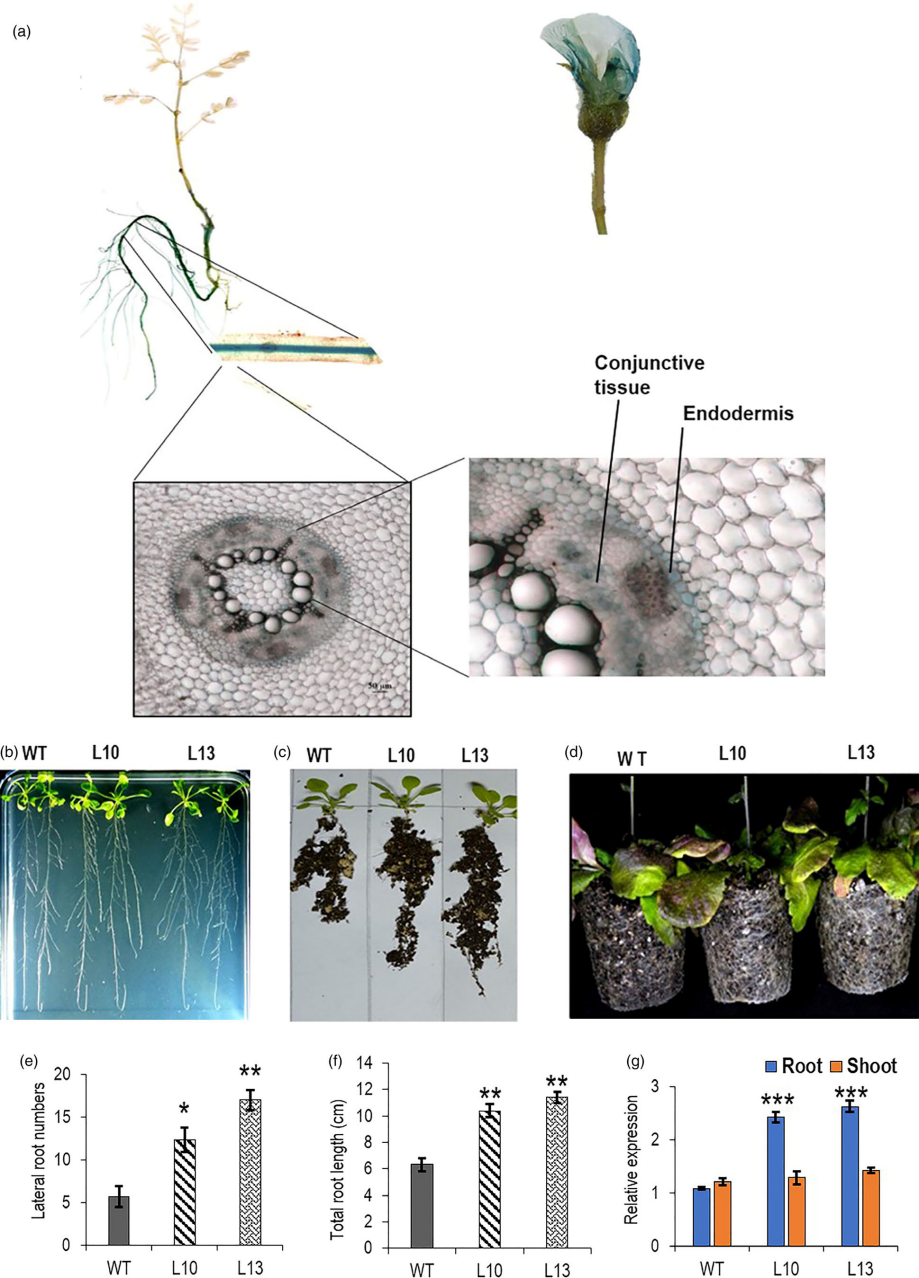
Tissue‐specific expression of *CaWRKY31* promoter and phenotypic analysis of *W31::CKX6*‐expressing transgenic *Arabidopsis* lines. (a) Localization of GUS activity in various tissues of *pCaWRKY31::GUS*‐expressing transgenic chickpea. Cell type showing GUS activity is shown. (b and c) Root phenotype of 14 dpg old *W31::CKX6* T3 homozygous lines (L10 & L13) grown in MS medium and soil. (d) Comparison of root network of 50 dpg old pot‐grown WT and transgenic *Arabidopsis* lines. (e and f) Lateral root numbers and total root length of 14 dpg old WT and two *W31::CKX6* lines grown in MS medium. Results of three replicates (*n* = 5) are shown. (g) Relative expression of *CaCKX6* in the root and shoot of 14 dpg old transgenic lines as compared to the WT *Arabidopsis*. *ACTIN2* (At3g18780) was used as internal control for RT‐qPCR. Bars represent means ± SE. Asterisks indicate statistically significant differences from WT determined using two‐tailed Student's *t*‐test (* for *P* < 0.05; ** for *P* < 0.01, *** for *P* < 0.001).

Previously, *AtWRKY6* promoter was used to direct root‐specific expression of *AtCKX1* (Werner *et al.*, 2010). The chickpea WRKY protein that showed the highest sequence homology with *AtWRKY6* has been annotated as *CaWRKY31* (XP_004507670.1). We have used the promoter of the corresponding gene (XM_004507613.3/ LOC101494311) following transcriptome‐based tissue‐specific expression analysis (Garg *et al.*, 2011; Figure S3a) for directing *CaCKX6* expression in root. *CaWRKY31* expressed primarily in the root of 10 day post‐germination (dpg) old chickpea seedling as observed by RT‐qPCR (Figure S3b and c). To investigate *in planta* expression pattern of *CaWRKY31* promoter in chickpea, an efficient chickpea transformation protocol was established by modifying published methods (Bhatnagar‐Mathur *et al.*, 2009; Chakraborti *et al.*, 2006; Jayanand *et al.*, 2003; Sarmah *et al.*, 2004; Sharma *et al.*, 2006). A 1500 bp genomic DNA fragment (Appendix S1) from *CaWRKY31* promoter including the 5′‐upstream activating sequence (5′UAS) was used to direct expression of reporter protein β‐glucuronidase (GUS) to monitor its tissue‐specific expression in chickpea. The transformed soil‐grown 6 dpg old chickpea plants showed expression of the reporter protein activity only in the root (both primary and the lateral root). Transverse section detected GUS activity in the endodermal layer and in some cells, in the conjunctive tissue of the root vascular system. In the matured plants (120 dpg old), in addition to root, flower showed a weak GUS activity, whereas the reporter protein activity was not detected in leaf and stem in the matured plant (Figure 2a).

*CaCKX6* coding sequence (CDS) was inserted under *CaWRKY31* promoter (*W31::CaCKX6*) to direct root‐specific expression of the gene in *Arabidopsis*. Significant increase in lateral root number was observed in 14 dpg old seedlings vertically grown in MS media. Pot‐grown transgenic lines showed higher soil‐holding capacity, enhanced root network at 14 and 50 dpg growth stages (Figure 2b–f). Expression of *CaCKX6* was confined to root only (Figure 2g). Shoot phenotypes such as rosette diameter, silique and seed number of the transgenic *Arabidopsis* lines were similar to those of the wild‐type plants, except the bolt height, which was significantly higher in the transgenic lines (Figure S4a–e).

### *CaWRKY31* promoter‐driven *CaCKX6* expression in chickpea caused enlarged root system, high CKX activity in root and an increase in seed yield

**Figure 3:**
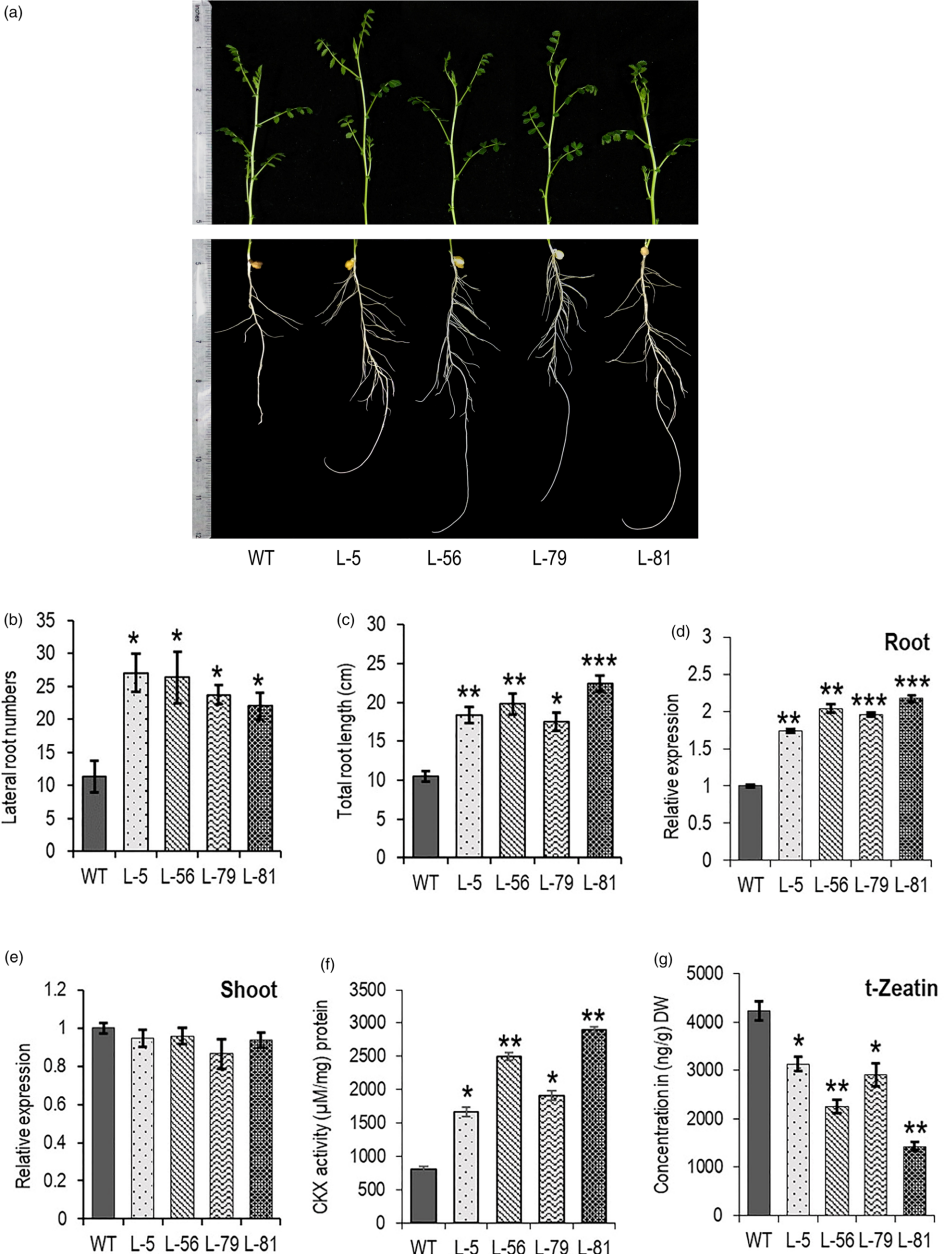
Root‐specific expression of *CaCKX6* alters the root traits and CKX activity in transgenic chickpea plants. (a–c) Root phenotype, lateral root numbers and root length of 10 dpg old WT (var. Pusa 362) and transgenic chickpea lines. (d and e) Relative expression of *CaCKX6* in root and shoot of 30 dpg old transgenic chickpea plants. Four independent transgenic lines were used, and their numbers are mentioned in the X‐axis (L‐5, L‐56, L‐79 & L‐81). RT‐qPCR was performed using three biological replicates, and *CaEF‐1α* (NM_001365163.1) was used as internal control for RT‐qPCR. (f) CKX enzyme activity in roots of 30 dpg old soil‐grown wild‐type (WT) and transgenic chickpea lines was determined using N^6^‐isopentenyladenine (2‐iP) as substrate. Three biological replicates (a pool of five plants) were analysed for each line. (g) trans‐Zeatin *t*‐Zeatin concentration in roots of WT (var. Pusa 362) and transgenic chickpea *t*‐Zeatin contents were quantified in 30 dpg old soil‐grown chickpea roots by UPLC‐coupled Orbitrap mass spectrometer. The values were measured using a standard curve. Three biological replicates were analysed for each line. Bars represent means ± SE. DW denotes dry weight. Asterisks indicate significant differences from the WT as determined by two‐tailed Student's *t*‐test (* for *P* < 0.05; ** for *P* < 0.01; *** for *P* < 0.001).

T_4_ transgenic chickpea plants were generated with *W31::CaCKX6* construct and chickpea cultivar Pusa 362 (IC296139). Detailed phenotypic and biochemical analyses were performed with four independent homozygous transgenic lines. Integration of single copy transgene in the genomic DNA was verified by Southern blot using a 196‐base probe designed with the sequence from the junction between *CaWRKY31* 5’UAS and *CaCKX6* CDS (Figure S5, Appendix S2). Chickpea transgenic plants followed the similar phenotypes of transgenic *Arabidopsis* and showed up to 1.8‐fold increase in root length and lateral root numbers in 10 dpg stage when grown in soilrite pots in controlled growth chamber (Figure 3a–c). A significant increase (1.7–2.2‐fold) in the expression of *CaCKX6* was observed by RT‐qPCR in the root, while expression of the transgene in the shoot was not detected (Figure 3d–e). CKX activity was assessed in soil‐grown 30 dpg plants, and an increase of 2.1–3.7‐fold over the untransformed plant was observed only in the root, while CKX activity in the shoot tissue (not shown) was not altered (Figure 3f). A significant reduction in cytokinin metabolite mostly, the *trans*‐Zeatin (tZ), by higher *CKX* expression was reported before (Ramireddy *et al.*, 2018a). A decrease in tZ content by 25%–66% as compared to the untransformed plant was observed in the roots of transformed chickpea lines (Figure 3g).

**Figure 4:**
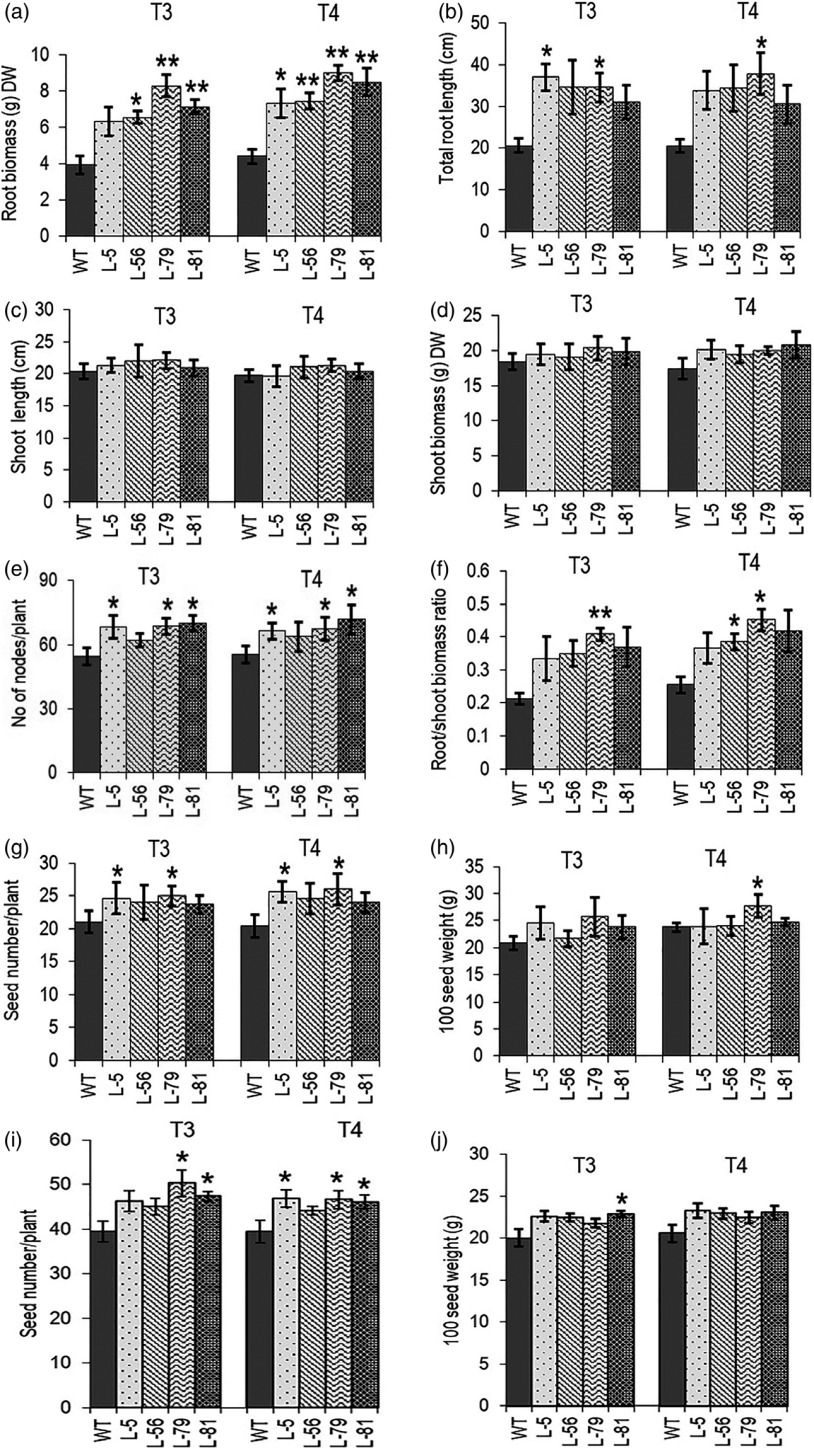
Characterization of various agronomic parameters in transgenic chickpea lines expressing *W31::CKX6*. (a) Root biomass (gram dry weight). (b) Total root length (cm). (c) Shoot length (cm) and (d) Shoot biomass (gram dry weight). (e) Number of nodes per plant. (f) Root‐to‐shoot biomass ratio. (g) Total seed number per plant. (h) 100 seed weight (gram). (I and j) Seed number per plant and 100 seed weight (gram) in net house soil bed grown plants. Bars represent means ± SE. DW denotes dry weight. Asterisks indicate significant differences from the WT as determined by two‐tailed Student's *t*‐test (*for *P* < 0.05; ** for *P* < 0.01; *** for *P* < 0.001).

The transgenic chickpea lines showed an average delay of fifteen days in maturity (155 days as compared to 140 days for the control plants). According to the quantitative phenotyping data of the transgenic chickpea lines grown in soil pots (single plant per pot, five plants per line) in controlled growth chamber (22–24 °C/60%RH/10 h light) for two years (T3 and T4), the biomass of the soil‐grown matured roots of transgenic lines showed 1.5‐ to 2‐fold increase in transgenic plants as compared to the control plant. Total root length increased by 1.5‐ to 1.85‐fold (Figure 4a and b). Plant heights of the transgenic lines did not show much change; however, average shoot biomass of five plants of each line showed an increase by up to 20% (Figure 4c and d; Figure S6a). Higher shoot biomass without changes in plant height was observed due to an increase in the number of nodes and secondary branches in the soil‐grown matured plants (Figure 4e). Root‐to‐shoot biomass ratio was increased by up to 1.7‐fold in transgenic chickpea lines (Figure 4f). All transgenic lines and the control plants started flowering at around 58 days in controlled growth conditions. Average seed number per plant showed 20%–25% increase as per two‐year growth data without significant variation in 100 seed weight (Figure 4g and h). These lines (twenty plants per line) were further evaluated for seed yield in a small netted soil bed covered with a transparent poly‐roof during mid‐October to mid‐March for two generations. Average seed number per plant in this condition also showed a similar increase of 15%–25% in transgenic lines. Although the average seed yield of every transgenic line was always higher than the control line, variation in seed numbers between the individual plants of a line sometimes resulted in low statistical significance (Figure 4i and j). Therefore, a larger multi‐year randomized field trial and assessment of yield per plot are required for field‐level evaluation of seed yield parameters.

### Higher CKX activity in root did not alter root nodulation and N_2_‐fixation

**Figure 5:**
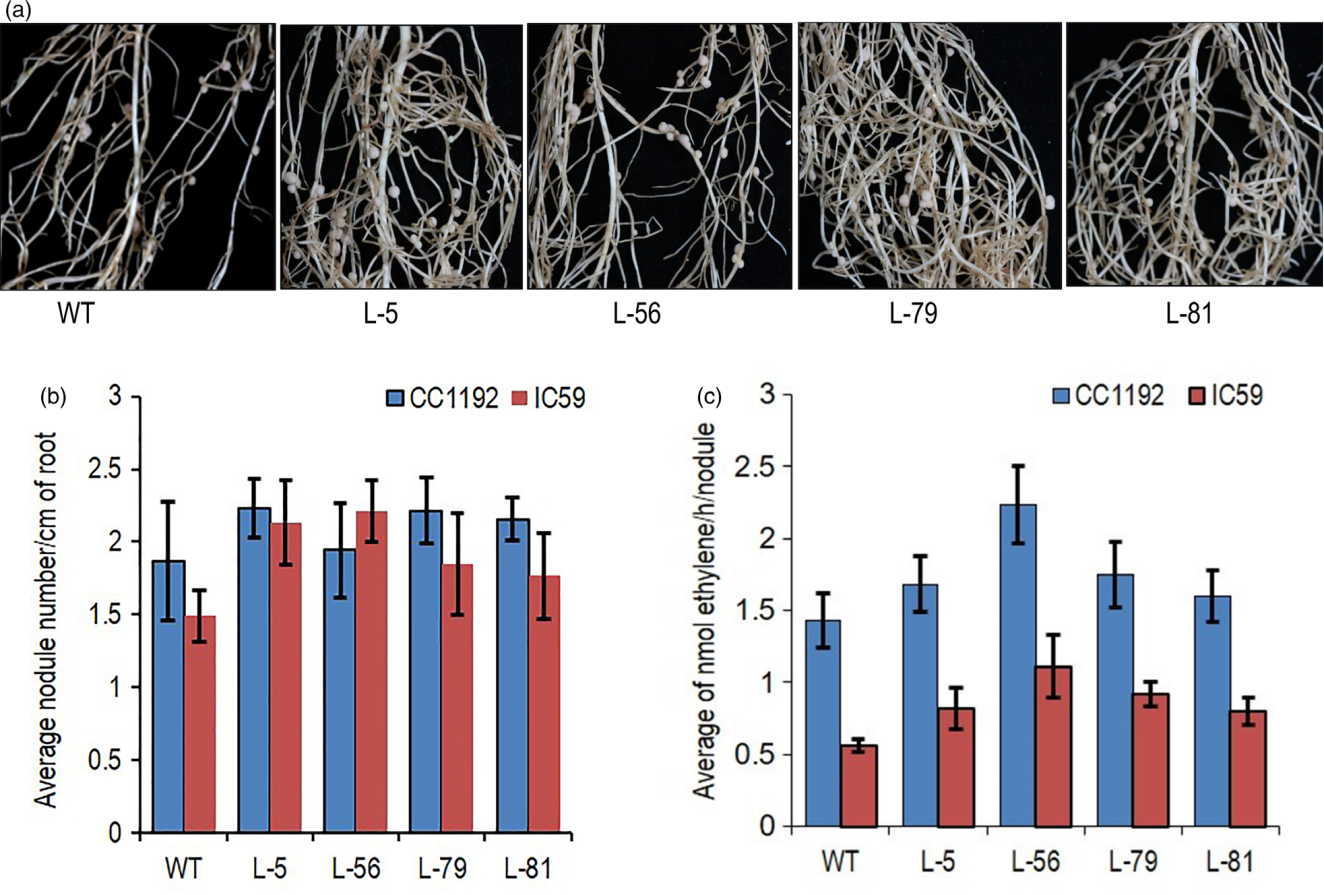
Evaluation of nodule formation frequency and nitrogen‐fixing efficiency in *CaCKX6* transgenic plants. (a) WT (var. Pusa 362) and transgenic chickpea lines showing nodule formation. (b) Average nodule number per cm of sensitive zone of root in chickpea lines formed after inoculation of two bacterial strains of the *Mesorhizobium ciceri.* The names of the strains are mentioned in the figure. (c) Average ethylene production per nodule per hour in the chickpea lines as determined by the acetylene reduction assay to evaluate nitrogen‐fixing efficiency after inoculation of two bacterial strains.

Root nodules are primarily found in leguminous plants, which form symbiotic relations with single‐celled Gram‐negative nitrogen‐fixing bacteria collectively called rhizobia. Grain and forage legumes provide 33% of dietary nitrogen requirement, apart from being a natural fertilizer (Graham and Vance, 2003). Nodule formation frequency was tested in transgenic chickpea roots by inoculating two different bacterial species (*M. ciceri* IC59 and *M. ciceri* CC1192) (Figure 5a). Nodule numbers per centimetre (cm) of root length was counted in the same root nodule forming zones of the plants. Average nodule numbers per cm of root from five plants of each line showed no significant alteration in nodulation frequency by both the bacteria (Figure 5b). The visible red colour of the nodules indicated the presence of leghaemoglobin (Figure S7a). Nitrogen‐fixing capacity of the whole root with nodules was tested by acetylene reduction assay. Transgenic lines produced an equivalent amount of ethylene suggesting root‐specific cytokinin degradation in the transgenic chickpea lines did not affect N_2_‐fixation in chickpea (Figure 5c). Average seed protein contents of the transgenic lines also did not show any significant changes as compared to the wild‐type plants (Figure S7b).

### Root‐specific expression of CaCKX6 enhances mineral contents in seeds

**Figure 6:**
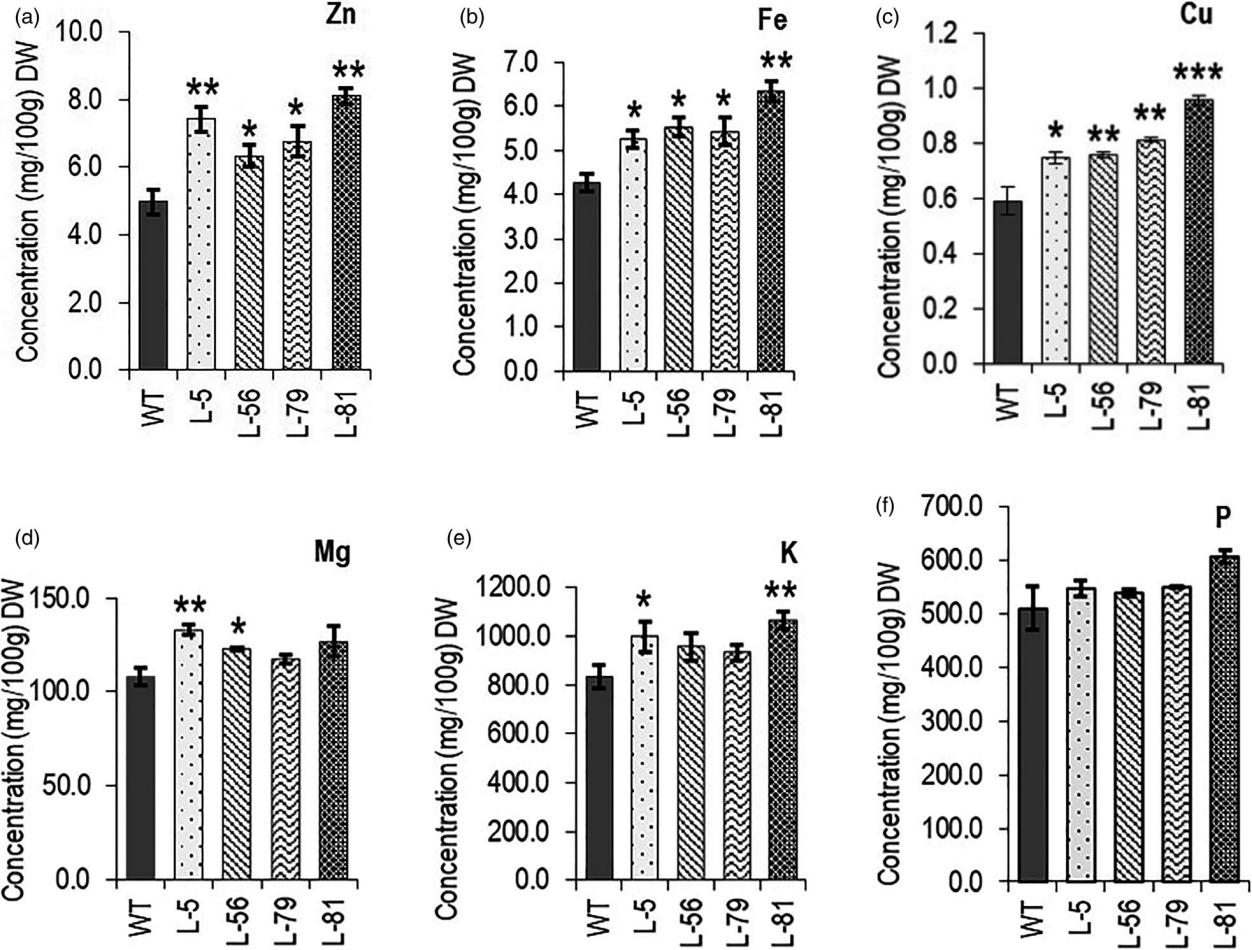
Evaluation of mineral content in 100 gm seed dry weight of the chickpea lines. Relative changes were observed in transgenic lines compared to wild‐type plants as determined by ICP‐MS. The minerals are mentioned in the respective figures. (a) Zinc (Zn). (b) Iron (Fe). (c) Copper (Cu). (d) Magnesium (Mg). (e) Potassium (K). (f) Phosphorus (P). Three biological replicates for each genotype were analysed. Bars represent means ± SE. DW denotes dry weight. Asterisks indicate significant differences from the WT as determined by two‐tailed Student's *t*‐test (* for *P* < 0.05; ** for *P* < 0.01, *** for *P* < 0.001).

Elevated zinc level has been reported in cytokinin‐deficient plants. It was recently reported that transgenic barley plants expressing a CKX gene in their roots form a larger root system and accumulate a higher concentration of Zn in their grains when grown under greenhouse conditions (Ramireddy *et al.*, 2018b). *OsCKX4*‐overexpressing cytokinin‐deficient rice lines showed increased zinc level in seeds (Gao *et al.*, 2019). To investigate the possibility of an enhanced mineral acquisition by the robust root system, the concentration of six different elements (zinc, iron, copper, phosphorus, magnesium and potassium) was measured in seeds. Seeds of transgenic lines showed higher concentration of zinc (27%–62%), copper (26%–61%), iron (22%–48%), magnesium (13%–22%), potassium (11%–27%) and phosphorus (5%–19%) (Figure 6a–f).

### Transgenic chickpea plants showed more drought tolerance

**Figure 7:**
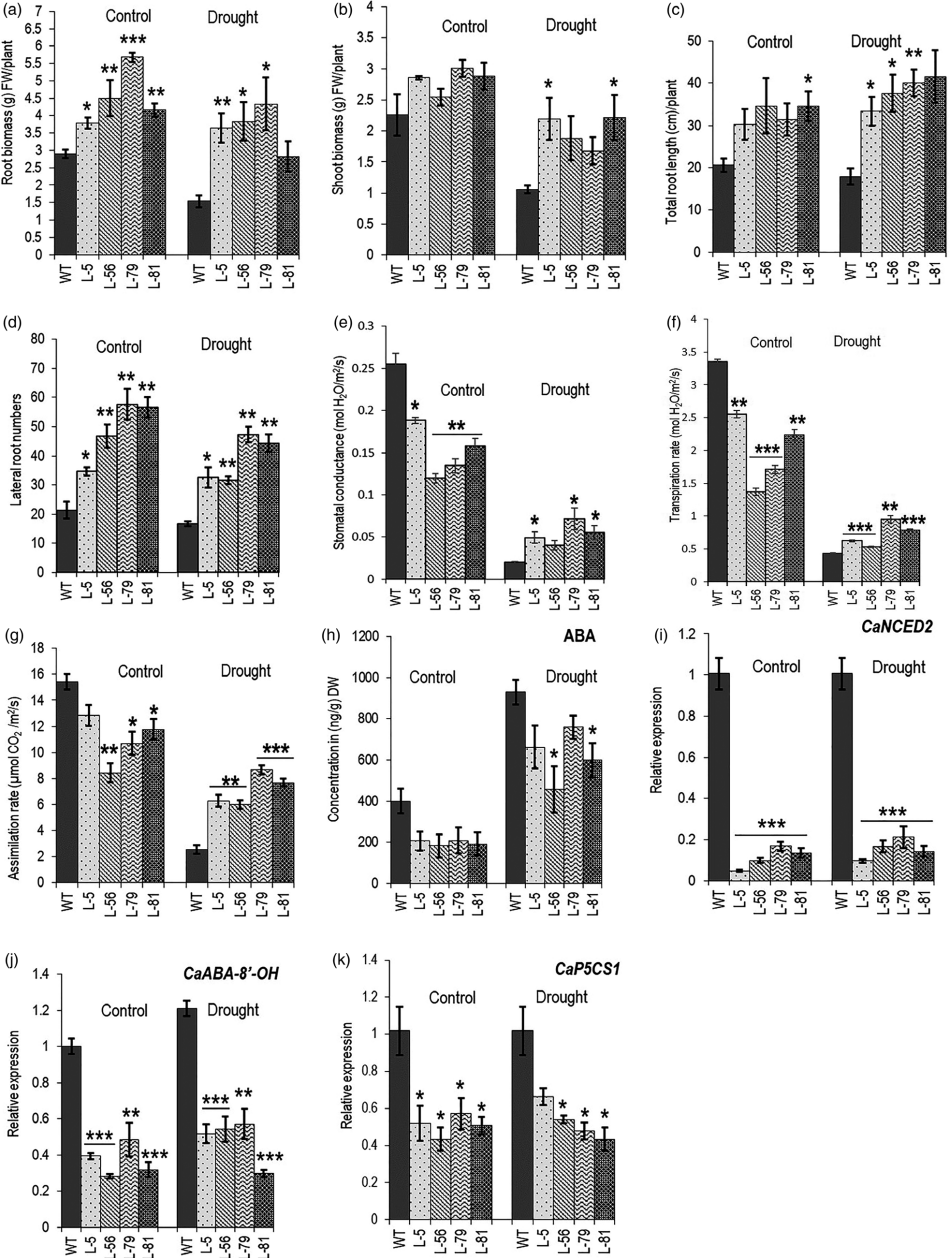
Effect of root‐specific expression of *CaCKX6* on drought tolerance and expression of ABA‐related genes in chickpea. (a–d) Measurement of agronomic parameters in WT and transgenic chickpea lines under drought stress. 14 dpg old chickpea plants growing in soil: sand (1:1/v:v) in PVC pipes were subjected to drought by withholding irrigation for 40 days. The measurements were taken at the end of the experiment. The plants in the control conditions were irrigated evenly every 3 days. Measurements of five plants for each line are presented. (e–g) Measurement of physiological parameters in the same plants as mentioned above after 20 days of withholding irrigation. The third leaf from the top was used for measuring physiological parameters. Relative soil moisture content of the pot is shown in Figure S8a. (h) ABA concentrations in the root of the chickpea lines under control and drought conditions. The drought treatment was given as mentioned before. ABA concentration was measured in one gram of dry root sample. Relative expression levels in the root of WT and transgenic chickpea lines of a gene involved in ABA synthesis (*CaNCED2*) (i); a gene involved in ABA degradation (*CaABA‐8’‐OH*) (j); and in the Pro synthesis gene (*CaP5CS1*) (k). Total RNA was extracted from root tissue of WT and transgenic plants grown under control and drought stress conditions. Bars represent means ± SE. FW denotes fresh weight. Asterisks indicate significant differences from the WT as determined by two‐tailed Student's *t*‐test (* for *P* < 0.05; ** for *P* < 0.01, *** for *P* < 0.001).

Chickpea is traditionally grown in marginal lands in the semi‐arid tropics in India, West Asia and North Africa as a spring crop and is dependent on residual soil moisture. Apart from diseases, drought is the most important constraint of chickpea yield. The crop is sown in the winter to avoid disease and, therefore, faces drought during the flowering stage resulting in yield loss (Devasirvatham and Tan, 2018). To assess long‐term drought tolerance, *W31::CaCKX6* chickpea lines along with the control plants (five plants of each line) were subjected to progressive drought stress by withholding irrigation to mimic natural drought. Seeds were sown in PVC pipes (50 cm height/ 20 cm diameter) containing soil and sand mixture (50:50/v:v) having a relative soil moisture content of 14%–17% throughout the pipe in growth chamber maintained with parameters mentioned above. A similar relative soil moisture level was maintained in one set of samples throughout the experiment by regular irrigation to maintain growth under control conditions. Irrigation to the other set of samples was withheld from 14 dpg onwards for further 40 days. Relative soil moisture content was measured at 6 cm and 25 cm depth. It decreased slowly and progressively to 3.5%–4.5% at 6 cm depth and to ~6.5% at 25 cm depth at 40th day, after irrigation was stopped (Figure S8a). A set of five plants of control line and each of four transgenic lines were grown and maintained at control conditions, and another set was subjected to drought treatment, as described above. As observed in the case of cowpea (Maia *et al.*, 2013), the average total root length of all the lines increased under drought, probably in search of soil moisture. Shoot biomass decreased significantly as expected. Interestingly, average root biomass also decreased despite an increase in root length after the drought. A decrease in the lateral root number might be one of the reasons for decreased root biomass (Figure 7a–d). The decrease in relative water content (RWC) of the leaves is considered as one of the physiological markers of drought stress. RWC of third and fourth leaves from the top of the plants of same age was measured under control and 40 day‐drought conditions. The relative decrease in RWC in the wild‐type plants was more than those of the transgenic lines (Figure S8b). Stomatal conductance, CO_2_ assimilation rate and transpiration rate of the third leaf from the top of all the lines showed a decrease in drought; however, decrease in these parameters was significantly less in the transgenic lines. While the decrease in the stomatal conductance, transpiration rate and CO_2_ assimilation rate in the control plants was 90%, 87% and 83%, respectively, the transgenic lines showed a decrease of 46%–73% for stomatal conductance, 43%–73% for transpiration rate and 18%–51% in CO_2_ assimilation rate (Figure 7e–g). The result with this limited number of test plants in control condition requires field‐level verification.

A lower abscisic acid (ABA) level was reported in the *AtCKX1‐4* – overexpressing *Arabidopsis* (Nishiyama *et al.*, 2011). Basal abscisic acid (ABA) concentrations in roots of the transgenic lines were significantly lower from the control plants. ABA level increased under water‐limited conditions in both the control and transgenic lines; however, the ABA level in the transgenic roots remained lower than the untransformed plants, most likely due to longer root in the transgenic lines and higher moisture content deep in the soil (Figure 7h). Expression levels of ABA biosynthesis gene, 9‐cis‐EPOXYCAROTENOID DIOXYGENASE2 (*CaNCED2*) and a key ABA‐degrading gene, ABA‐8′‐HYDROXYLASE (*CaABA‐8′‐OH*) were also much lower in transgenic chickpea plants as compared to the wild type. However, a maximum of 20‐fold reduction in *CaNCED2* level as opposed to a threefold reduction in *CaABA‐8’‐OH* level was observed under control conditions, supporting an overall reduced ABA accumulation in the transgenic lines (Figure 7i and j). Lesser stress conditions in the transgenic lines were also observed by lower expression of proline biosynthesis gene *P5CS1* (Δ1‐pyrroline‐5‐carboxylate synthetase1) (Verslues and Sharma, 2010) (Figure 7k).

## Discussion

Over two decades, cytokinin oxidase/dehydrogenase (CKX) has been used extensively as a tool for studying the role of cytokinin in plant development in *Arabidopsis*. Seven CKX isoforms of *Arabidopsis* showed differential tissue‐specific expression, subcellular protein localization and substrate specificity (Werner *et al.*, 2003). However, overexpression of various *AtCKX* in *Arabidopsis* under constitutive CaMV35S promoter was reported to develop similar qualitative phenotype with stunted shoot growth and enhanced root formation (Werner *et al.*, 2008; Werner *et al.*, 2003). Our result showed overexpression of chickpea *CKX6* in *Arabidopsis* produced a similar previously reported phenotypic effect of *AtCKX6* overexpression in *Arabidopsis*. Ca*CKX6* protein is twenty‐one amino acid longer than *AtCKX6* in the N‐terminus. However, the similar phenotypic effect was probably due to their high sequence similarity (81% similarity, 62% identity). This result suggested that orthologous *CKX* genes can function similarly across the species.

Expression of *W31::CaCKX6* in chickpea root enhanced root biomass along with the increase in shoot biomass and yield. The results suggested that more carbon partitioning in the root was not at the cost of the shoot. This observation raised a long‐debated issue of carbon partitioning between vegetative and reproductive growth, and between shoot and root. A carbon‐centric view was theorized that proposed a rate‐limiting role of net carbon assimilation in plant growth and net primary production. However, while accepting the prime role of photosynthesis‐derived carbohydrate as the building block of plant biomass, carbon can be only invested in biomass if other essential elements are available optimally. It is generally accepted that nitrogen is the most important limiting soil nutrient and the other elements such as phosphorus, potassium, magnesium and molybdenum are critical for growth. As under natural growth conditions, photosynthesis mostly operates below the saturated light, and at a lower rate than the maximum capacity of the leaves, net primary production is generally not constrained by the capacity of leaves to assimilate carbon dioxide. This raised a parallel argument emphasizing the role of environment on sink activity as the limiting factor for tissue growth in which demand of assimilate usage, in addition to the assimilation, is ultimately responsible for net primary production at favourable growth conditions where the plant is not resource‐starved (Körner, 2015; Marcelis, 1996; Sonnewald and Fernie, 2018). However, in resource‐starved conditions, the larger root system of transgenic lines might have provided more resources to the source tissue for higher carbon assimilation.

The strategy of enhancing root biomass by decreasing cytokinin level in the root has been tested so far, in *Arabidopsis*, tobacco and barley, plant species having determinate growth habit (Ramireddy *et al.*, 2018a; Werner *et al.*, 2010; Werner and Schmulling, 2009). In those plants, increased root biomass and mineral content did not affect vegetative and reproductive growth of the shoot. In contrast, we have observed a limited but significant increase in shoot biomass and seed yield in transgenic chickpea lines. Cultivated chickpea genotypes show an indeterminate growth habit. In these genotypes, the terminal shoot apical meristem remains in a vegetative state during the production of new nodes and leaves, as well as during the production of inflorescence meristems that generate axillary floral meristems. Hence, these genotypes continue to grow in stem length, flower and set pods as long as environment and resources permit (Bradley *et al.*, 1997; Hegde, 2011; Tiana *et al.*, 2010). We argue that the increase in vegetative and reproductive growth of shoot in these transgenic chickpea lines was due to the acquisition of more resources from the soil by the larger root network that increased longevity of the plant. *Arabidopsis* with determinate growth pattern did not show a similar increase in shoot growth when transformed with the same construct.

Chickpea lines expressing *W31::CaCKX6* showed improved drought tolerance showing higher RWC in leaves as compared to the wild‐type plants. As the relative moisture content was higher deep in the soil, the acquisition of more resources by a deeper root system might be one of the reasons for improved tolerance. Additionally, cytokinin‐deficient plants were reported to exhibit stress‐tolerant phenotype associated with cell membrane integrity and ABA hypersensitivity. Under normal conditions, cytokinin deficiency was reported to cause a significant reduction in endogenous ABA level (Nishiyama *et al.*, 2011). We observed a similar reduced ABA level in cytokinin‐deficient chickpea roots under control conditions. Although ABA level increased in both wild‐type and transgenic chickpea lines under drought, ABA level in the transgenic lines was 18%–50% less than that of the wild type. Higher leaf RWC together with lower ABA level might have contributed to higher carbon assimilation under long‐term drought conditions.

Zinc and iron are critical for human nutrition. Therefore, enrichment of zinc and iron is a desirable trait for crops. Maize gene encoding zinc‐ and iron‐regulated transporter‐like protein (ZmZIP5) was able to functionally complement zinc‐uptake double mutant and iron‐uptake double mutant in yeast. Higher zinc and iron contents were observed in *ZmZIP5*‐overexpressing maize line (Li *et al.*, 2019a). Cytokinin plays a crucial role in zinc uptake by regulating zinc transporter and chelators in plant cells (Gao *et al.*, 2019; Ramireddy *et al.*, 2018b; Sakakibara, 2006). After entry into the root, water and nutrients move radially to enter the central vascular system after crossing a restrictive endodermal cell layer. Some of the endodermal cells are less suberized and are known as passage cells that allow diffusion of water and nutrient towards vasculature. External application of cytokinin in *Arabidopsis* at a concentration that did not affect root growth decreased passage cell numbers, whereas expression of cytokinin‐signalling suppressor increased the number of passage cells by decreasing endodermal suberization. Further, ABA strongly promotes endodermal suberization and decreased passage cell numbers (Andersen *et al.*, 2018). Lower cytokinin and ABA levels in the roots of the transgenic lines might have helped the loading of nutrients in the vascular system. However, the data are from pot grown plants under control condition and need to be further confirmed by more field trials and at multiple locations with different soil zinc content.

Despite an overall decrease in cytokinin level in transgenic chickpea roots, we did not observe any decrease in nodule number or nitrogen fixation. Rather, the average nodule number per centimetre was higher although, not significantly. Role of cytokinin in nodule initiation and development might be positive or negative and may vary with tissue, legume species and time after bacterial inoculation (Gamas *et al.*, 2017). The number of nodules can increase or decrease under reduced cytokinin level, depending on the tissue specificity of the perturbation (Jardinaud *et al.*, 2016; Lohar *et al.*, 2004). Expression levels of *Lotus japonicus CKX3* gene (*LjCKX3*) and two *M. truncatula CKX* genes (*Medtr4g126150 and Medtr2g039410*) were induced one day after inoculation with rhizobial bacteria (Ariel *et al.*, 2012; Held *et al.*, 2008; van Zeijl *et al.*, 2015). *LjCKX3* expresses in the pericycle cells adjacent to protoxylem. Two independent *L*. *japonicus ckx3* mutant lines showed higher levels of tZ base and riboside in normal growth conditions and after bacterial inoculation, and exhibited reduced root growth and nodule number (Reid *et al.*, 2016), indicating controlled cytokinin level manipulation near pericycle layer impacts nodule formation. *CaWRKY31* promoter was mainly active in the endodermis, and the cytokinin level of pericycle in *W31:CaCXK6* lines might be enough to support optimum nodulation. Further, as in *L. japonicus*, lower ABA level could have been a reason for maintaining nodule number in these plants (Tominaga *et al.*, 2010).

To conclude, expression of an *Arabidopsis CKX6* ortholog of chickpea in the root endodermal cells of chickpea produced a robust increase in root network and improved tolerance to long‐term drought without any apparent negative effect on the shoot. Chickpea is mostly grown in semi‐arid regions during the post‐monsoon season and face terminal drought during flowering, causing a huge yield penalty (Kashiwagi *et al.*, 2006; Samarah and Alqudah, 2011). Enhancing the root network by local manipulation of cytokinin level might be an effective approach along with conventional breeding to alleviate yield loss in chickpea and other indeterminate legume crops.

## Experimental procedures

### Description of vector construction for molecular cloning

*CaCKX6* cDNA was amplified from total chickpea (Pusa 362) mRNA by reverse transcription (RT)‐PCR (Verso cDNA synthesis Kit, Thermo Fisher Scientific, Carlsbad, CA) using the gene‐specific forward and reverse primers (CaCKX6_gty_F and CaCKX6_gty_R) mentioned in Table S1 and was cloned in Gateway entry plasmid pENTR using D‐TOPO Cloning Kit (Thermo Fisher Scientific) following manufacturer’s protocol. Subsequently, the insert was transferred to Gateway binary plasmid pGWB2 for expression under CaMV35S promoter through LR clonase (Thermo Fisher Scientific) reaction. For subcellular localization of CaCKX6, M13_F and M13_R were used to amplify PCR product from pENTR clone of *CaCKX6* and cloned to pEG101 Gateway binary vector through LR clonase reaction. Further, 1.5 kb promoter region of *CaWRKY31* gene was amplified from genomic DNA using specific primers (CapWRKY31_gty_F and CapWRKY31_gty_R) mentioned in Table S1 and cloned first in pENTR D‐TOPO vector and then in binary plasmid pGWB3 together fused to *uidA* reporter gene. To express *CaCKX6* gene under CaWRKY31 promoter, CaWRKY31 promoter was amplified using genomic DNA using primers (CapWRKY31_F and CapWRKY31_R) and cloned in pBI101.2 with Xba1 and Sma1 restriction site. *CaCKX6* cDNA was amplified from total mRNA by RT‐PCR using primers (CaCKX6_F and CaCKX6_R) and cloned after CaWRKY31 promoter between Sma1 and Sac1 sites in pBI 101.2 binary vector.

### Plant materials, transformation and growth conditions

*Arabidopsis thaliana* (ecotype Columbia‐0) plants were grown on soil in a growth chamber with 80 µmol/m^2^/s light at 22–24 °C with a photoperiod (16 h light) for 4–5 weeks for normal growth. The floral dip method was used to generate *Arabidopsis* transgenic plants (Clough and Bent, 1998). The presence of the transgene and its expression were confirmed by PCR and reverse transcription‐PCR (RT‐PCR), respectively. Chickpea plants were grown in the plant growth chamber at 22–24 °C and 60% of humidity with 10‐h light period with an intensity of 250 μmol/m^2^/s. Same soil lot was used for the comparative studies. No additional fertilizer or nutrient was used in the soil. Equal irrigation when required was done with RO water. For chickpea plants, the desi cultivar Pusa 362 (IC296139) was used for transformation. Transgenic plants were generated using *Agrobacterium tumefaciens* strain‐mediated transformation of single cotyledon and half embryo as the explant (Bhatnagar‐Mathur *et al.*, 2009; Chakraborti *et al.*, 2006). For primary culture growth, *Agrobacterium* culture was prepared in Luria–Bertani (LB) medium containing 25 mg/L rifampicin and 50 mg/L kanamycin. This culture was shaken (200 r.p.m.) overnight at 28 °C. The secondary culture was prepared by using 1% inoculum of the primary culture containing 25 mg/L rifampicin and 50 mg/L kanamycin and was shaken as a primary culture under the same temperature and time regime. Finally, 100 μm acetosyringone was added to the culture before the transformation. Explants were dipped in the culture for 20 min. Co‐cultivation medium (MS medium) was used for further incubation of explants for 48 h in dark at 22 °C. After 48 h, these explants were washed with autoclaved MilliQ water containing cefotaxime 250 mg/L to remove the excess bacterial cells. These explants were further kept on shoot induction medium 1 and 2 containing kanamycin as a selection marker (150 mg/L) for the next 24 days. After completion of sub‐culturing and selection of healthy shoots, shoot elongation medium (cefotaxime 250 mg/L and kanamycin 100 mg/L) was used for further elongation of shoots. After 12 days, healthy and selected shoots were picked and maintained on normal MS medium (cefotaxime 250 mg/L, only) to stop bacterial contamination. These shoots were further used for micrografting and establishment of mature plants. Agrobacterium strain GV3101 containing the binary vector pBI101.2 was used for all transformations in chickpea and *Arabidopsis*. Positive transformants were detected by genotyping and Southern blotting.

### Subcellular localization and fractionation of CaCKX6

The *35S::CaCKX6‐YFP* construct generated by Gateway Technology (Invitrogen) in the pEG101 binary vector was transformed into *Agrobacterium tumefaciens* (EHA105) and infiltrated in *N. benthamiana* leaves for transient expression. For agro‐infiltration, OD_600_ = 0.6 grown cell cultures were pelleted and resuspended in 10 mL of infiltration buffer (10 mm MES‐KOH, pH 5.6, 10 mm MgCl_2_, and 150 µm acetosyringone). The working suspensions were prepared by mixing the Agrobacterium culture harbouring the *35S::CaCKX6‐YFP* construct with an organelle marker for the plasma membrane (CD3‐1007 mCherry) and ER marker (CD3‐959 mCherry) (Nelson *et al.*, 2007) in a 1:1 ratio, incubated at room temperature for 3 h, and infiltrated onto the abaxial surface of fully developed leaves. Plants were kept for 2–3 days under growth room conditions (22 °C temperature, 16‐h light/8‐h dark photoperiod and 150 µmol/m^2^/s light intensity). Florescence was observed in confocal microscope Leica SP5 (Leica Microsystems) equipped with appropriate lasers (514–527 nm and 587–610 nm for YFP and mCherry, respectively). Fluorescence intensity scans were performed using the quantification tool of the Leica confocal software application (version 2.61). Subcellular fractionation of CaCKX6–yellow fluorescent protein (YFP) was performed by following Srivastava *et al.,* (2017) and Barua *et al.,* (2016). Thirty five micro grams of microsomal, cytosolic and plasma membrane fractions were separated by SDS‐PAGE followed by Western blot, and probed with anti‐green fluorescent protein (GFP) (Abcam, UK) antibody. The blotted membrane was probed with the antibodies against fraction‐specific marker proteins, anti‐Bip2 (microsomal fraction) and anti‐cytosolic fructose‐1,6‐bisphosphatase antibodies (cytosolic fraction), and anti‐plasma membrane H^+^ ATPase (plasma membrane fraction) (Agrisera, Sweden).

Experimental methods for histochemical GUS staining and protein localization and RNA isolation, RT‐qPCR, CKX activity assay and phytohormone assay are described in Appendix S3.

### Element measurements

For elemental measurements, 100 mg of dry chickpea seeds was crushed in liquid nitrogen. For determination of elemental concentrations, the dry samples were then digested with 7 mL nitric acid/perchloric acid mixture (4:1 v/v) at 100 °C for 2 h and 150 °C for 5 h. The acid‐digested samples were diluted to a final volume of 14 mL with ultrapure water. The metal concentrations were measured using inductively coupled plasma emission spectrometry (ICP‐MS; PerkinElmer Élan 9000‐USA). For better operating conditions, the ICP‐MS was adjusted to nebulizer gas flow 0.91 L/min, radio frequency (RF) 1200 W, lens voltage 1.6 V, cool gas 13.0 L/min and auxiliary gas 0.70 L/min (Jarapala *et al.*, 2014). Experiments were performed with at least three independent biological replicates.

### Root nodulation and acetylene reduction assay

Chickpea seeds were surface sterilized with 30% commercial bleach solution which contains active sodium hypochlorite 1.2% for 3 min. After this, the seeds were thoroughly washed with sterile water and placed on plates containing sterile wet filter paper. The plates were kept at 22 °C temperature in dark. After 2–3 days, the germinated seedlings were placed in the pots (11 cm long) containing growing substrate (a mixture of 3:1 vermiculite and fire clay balls, leca). The pots were placed in the growth chamber (200 µmole/m^2^/s light intensity; 16‐h light/8‐h dark cycles; 22 °C temperature; 40%–60% relative humidity). Two bacterial strains *Mesorhizobium ciceri* IC59 and *Mesorhizobium ciceri* CC1192 were grown at 28 °C in tryptone yeast broth (Esfahani *et al.*, 2014; Haskett *et al.*, 2016; Rupela and Sudarshana, 1990). The inoculum was prepared from *M. ciceri* IC59 and *M. ciceri* CC1192 culture of OD_600_ 0.7–0.9. The bacterial culture was harvested (centrifuged at 2900 *g* for 10 min) and diluted in nitrogen‐free B and D media (Broughton and Dilworth, 1971). Each pot was inoculated with 100 mL of this diluted OD_600_ = 0.03–0.05 bacterial culture. Acetylene reduction assay (ARA) was performed as described previously (Mandal and Sinharoy, 2018; Oke and Long, 1999). Ethylene production was measured by gas chromatography (Shimadzu GC‐2010 equipped with HP‐PLOT ‘S’ Al_2_O_3_ 50 m, 0.53 mm column (Agilent Technologies, 19095P‐S25).

### Phenotyping and photosynthetic assessments

For the study of root system architecture and shoot phenotype, four selected chickpea lines were sown in the equal‐sized pots filled with an equal weight of soil along with non‐transformed wild‐type control plants in plant growth chambers. A phenotypic evaluation was performed for shoot phenotype in mature plants. The dry mature plants (155 days old) were uprooted carefully and manually. After harvest, chickpea plants were separated into shoots and roots manually. Roots were carefully lifted from the soil pots and cautiously washed to remove bound soil particles. Before imaging, roots were spread out in a root‐positioning tray filled with water to minimize overlap. Afterwards, separated shoots and roots were dried at 80 °C for 72 h and the dry weight was recorded.

For root phenotyping of the chickpea plants in control and drought conditions, plants were grown in plant growth facility (PGF) using a cylinder culture method in PVC pipes of size 20 cm diameter, 50 cm tall in a randomized complete block design (RCBD) with five replicates. The soil was an equi‐mixture (mixing the equal amount of proportion) (v/v) of soil and sand. The soil was equilibrated with a soil water content to create normal chickpea growth conditions. In this condition, the soil is not fully saturated with water at sowing time. This mixture (soil and sand) was used so that excess water was not stored in soil and it would also help in easy extraction of roots from the soil. Additionally, in loose soil, the root length will be mostly governed by genetic potential avoiding environmental variations. Each pipe was then irrigated with 500 mL of water after 15 days of seed sowing to ensure uniform seedling emergence. Later, the plants were allowed to grow without watering for 40 days. Relative soil moisture was measured at 6 and 25 cm depth using soil moisture meter (Lutron, PMS‐714, Taiwan). Control plants were continuously irrigated to maintain normal moisture condition. Relative water content (RWC) was determined according to Turner (1981). Portable photosynthesis system LI‐6400 XT (LI‐COR Biosciences, Inc.) was used for measurement of photosynthesis‐related parameters. The instrument was stabilized for 30 min in the greenhouse where measurements were taken. The measurements were only taken once after the internal CO_2_ concentration had stabilized (2–3 min after insertion of the leaf into the measuring chamber). A constant supply of 400 ppm CO_2_ (flow rate 200 μmol/s) was provided by a CO_2_ cartridge at a photon flux density of 900 μmol/m^2^/s by a mixed red/blue LED light source present above the leaf chamber head. The net assimilation rate, internal CO_2_ concentration, stomatal conductance and transpiration rate were all recorded from four biological replicates and three technical replicates. All the parameters were recorded at the same time in the morning hours.

## Conflict of interest

The authors have no conflicts of interest to declare

## Author contributions

DC initiated, designed, conceived, coordinated the project and wrote the manuscripts. HK, SKG and VD raised transgenic plants and assisted manuscript preparation. DM, LP, NKS, NKV, MC, PM, AF, NPS and KS performed the supporting experiments. SS, NP and RS guided various experiments. All authors have read and approved the final manuscript.

## Supporting information


**Figure S1** Phylogenetic analysis of CKX proteins in *Arabidopsis*, *Medicago* and chickpea.
**Figure S2** Phenotypic effect of *CaCKX6* overexpression in *Arabidopsis.*
**Figure S3** Tissue‐specific expression of *CaWRKY31* gene in chickpea.
**Figure S4** Comparison of phenotypic parameters of WT and *W31::CKX6 Arabidopsis* plants.
**Figure S5** Southern blot hybridization of the chickpea transgenic events.
**Figure S6** Phenotypic observation of matured transgenic chickpea plants
**Figure S7** Nodule phenotype and total seed protein estimation in transgenic plants.
**Figure S8** Relative soil moisture content and leaf relative water content of control and drought‐treated chickpea plants.
**Table S1** List of primers used in this study.
**Appendix S1** *CaWRKY31* Promoter sequence.
**Appendix S2** Probe sequence for Southern blotting.
**Appendix S3** Histochemical GUS staining and protein localization, RNA isolation, RT‐qPCR, CKX activity assay and Phytohormone assay.
