# Supplementary material for: Root‐specific expression of chickpea cytokinin oxidase/dehydrogenase 6 leads to enhanced root growth, drought tolerance and yield without compromising nodulation
Source: Plant Biotechnol J. 2020 Sep 1;18(11):2225–40. doi: 10.1111/pbi.13378 (PMC7589355; doi:10.1111/pbi.13378)
Supplement: Supplementary file 1 — Figure S1 Phylogenetic analysis of CKX proteins in Arabidopsis, Medicago and chickpea. Figure S2 Phenotypic effect of CaCKX6 overexpression in Arabidopsis. Figure S3 Tissue‐specific expression of CaWRKY31 gene in chickpea. Figure S4 Comparison of phenotypic parameters of WT and W31::CKX6 Arabidopsis plants. Figure S5 Southern blot hybridization of the chickpea transgenic events. Figure S6 Phenotypic observation of matured transgenic chickpea plants Figure S7 Nodule phenotype and total seed protein estimation in transgenic plants. Figure S8 Relative soil moisture content and leaf relative water content of control and drought‐treated chickpea plants. Table S1 List of primers used in this study. Appendix S1 CaWRKY31 Promoter sequence. Appendix S2 Probe sequence for Southern blotting. Appendix S3 Histochemical GUS staining and protein localization, RNA isolation, RT‐qPCR, CKX activity assay and Phytohormone assay. [file PBI-18-2225-s001.pdf]

# Supplementary information

**Root-specific expression of chickpea Cytokinin oxidase/dehydrogenase 6 in chickpea leads to enhanced root growth, drought tolerance and yield without compromising nodulation**

**Khandal et al.**

(a)

```
AtCKX6_AT3G63440.1              --------------------MSYLHASLLRKRTMLIVRSFTILLLSCIAFKLAC-CF---    36
CaCKX6-like_XP_012574863.1      --------------------MKHHPISLFGQHNIFFLKFFIILFLSCIATRFNF-CL---    36
MtCKX6_XP_003605865.1           -------------------------MSFFGKQNILFLKGFMILFLSCIATRFNF-CL---    31
CaCKX6-like_XP_004514814.1      MLNFKLLHPLTTNNISSIKTFRYPSFSLVKQYNIMFIKGLMILFLSCITIRLNF-CI---    56
MtCKX6_XP_003599606.1           MFNFNLLHPLTTN-IYSIKNLKHPSFSLVSEYNILFIKGFMILFLSCITIRLNF-CI---    55
AtCKX1_AT2G41510.1              -------------------MGLTSSLRFHRQNNKTFLGIFMILVLSCIPGRTN-LCSNHS    40
CaCKX1_XP_004493454.1           --------------------MVLKLIGFSKHVIFSFIKTIILLILNFILHKADSGCNNSV    40
MtCKX1_XP_003625100.1           --------------------MVLKLVSFSKHVIFSFTKTIIILILSFILYKADSGCNNSI    40
                                                          :  .    :   : :*.*. *  :    *

AtCKX6_AT3G63440.1              ------------SSSISSLKALPLVGHLEFEHV--HHASKDFGNRYQLIPLAVLHPKSVS    82
CaCKX6-like_XP_012574863.1      ------------SNIPSSLKTLPLVGHLSFDELSLTKASRDFGNRYQYHPMAILYPNSVS    84
MtCKX6_XP_003605865.1           ------------SNNPFSLKTLPLEGHLSFDETSLNKASKDFGNRYQYHPMAVMYPKSVS    79
CaCKX6-like_XP_004514814.1      ------------SSIPSSLKSLPLEGHFSFDEFDLKKAARDFGNRYQSFPMTVLHPKSVS    104
MtCKX6_XP_003599606.1           ------------SSIPFSLKTLPLEGHFSFDELDLKNAAKDFGNRYQSHPMTVLHPKSVS    103
AtCKX1_AT2G41510.1              VSTPKELPSSNPSDIRSSLVSLDLEGYISFDDV--HNVAKDFGNRYQLPPLAILHPRSVF    98
CaCKX1_XP_004493454.1           T---TSLVQFPPNEVLISLQSQVLDGHLSLKDN--EDAAKDFGNIHHFPPLAVLHPKSVS    95
MtCKX1_XP_003625100.1           T---TSLVQISPYDVIISLQSQSFDGHLSLKDN--EDAAKDFGNIHHFPPLAVLHPKTVS    95
                                            .   ** :  : *::.:..    ..::**** ::  *:::::*.:*

AtCKX6_AT3G63440.1              DIASTIRHIWMMGTHSQLTVAARGRGHSLQGQAQTRHGIVIHMESLHPQKLQVYSVDSPA    142
CaCKX6-like_XP_012574863.1      DIANTIKHIWNMGHSSHLTVAARGHGHSLSGQAQTHGGIVINMESLKVYEMNVDVIGNDS    144
MtCKX6_XP_003605865.1           DIATTIKHIWHMGHSSHLTVAARGHGHSLQGQSQTHGGIVIKMESLKVPEMQIH-VGNSS    138
CaCKX6-like_XP_004514814.1      DISVTIKHVWNLGPNSQLTVAARGHGHSLQGQAQAHGGVVINMESLNKVDEMKVYAGGEF    164
MtCKX6_XP_003599606.1           DIAVTIKHIWNLGPSSHLTVAARGHGHSLQGQAQAEEGVVINMESLNV-EEIKVY-GGEF    161
AtCKX1_AT2G41510.1              DISSMMKHIVHLGSTSNLTVAARGHGHSLQGQALAHQGVVIKMESLRSPDIRIY--KGKQ    156
CaCKX1_XP_004493454.1           DISRTIKHVFEKGSDSELKIAARGHGHSLQGQSQAHEGLVIKMESLQGHEMKIH--SGEF    153
MtCKX1_XP_003625100.1           DISRTVKHIFEKGSDSELKVAARGHGHSLQGQAQAHQGLVIKMESLQSPEMKIQ--TGEF    153

AtCKX6_AT3G63440.1              PYVDVSGGELWINILHETLKYGLAPKSWTDYLHLTVGGTLSNAGISGQAFRHGPQISNVH    202
CaCKX6-like_XP_012574863.1      PYIDVSGGELWINILHESLRYGLTPRSWTDYLHLTVGGTLSNAGVSGQAFKHGPQISNVQ    204
MtCKX6_XP_003605865.1           PYVDVSGGELWINILHETLKYGLAPRSWTDYLHLTVGGTLSNAGVSGQAFKHGPQISNVQ    198
CaCKX6-like_XP_004514814.1      AYVDVSGGELWIKILHETLKYGLAPRSWTDYLHLTVGGTLSNAGVSGQAFRHGPQISNVL    224
MtCKX6_XP_003599606.1           PYVDVSGGELWIKILHETLKYGLAPRSWTDYLHLTVGGTLSNAGVSGQAFRHGPQISNVQ    221
AtCKX1_AT2G41510.1              PYVDVSGGEIWINILRETLKYGLSPKSWTDYLHLTVGGTLSNAGISGQAFKHGPQINNVY    216
CaCKX1_XP_004493454.1           PYVDVSGGELWINILHETLKHGLAPKSWTDYLHLTVGGTLSNAGISGQAFKHGPQINNIF    213
MtCKX1_XP_003625100.1           PFVDVSGGELWINILHETLKHGLAPKSWTDYLHLTVGGTLSNAGISGQAFRHGPQINNIF    213
                                 ::******:**:**:*:*::**:*:*****************:*****:*****.*:

AtCKX6_AT3G63440.1              QLEIVTGKGEILNCTKRQNSDLFNGVLGGLGQFGIITRARIALEPAPTMVKWIRVLYLDF    262
CaCKX6-like_XP_012574863.1      QLEIVTGTGEVVNCSKDQNGELFHSVLGGLGQFGIITRARILLEPAPSMVKWIRVLYSDF    264
MtCKX6_XP_003605865.1           QLEIVTGTGEVVNCSEKQNEELFQSVLGGLGQFGIITRARILLEPAPSMVKWIRVLYSDF    258
CaCKX6-like_XP_004514814.1      KMEIVTGTGEVVNCSKEENVELFYSVLGGLGQFGIITKARILLEPAPTMVKWIRVLYADF    284
MtCKX6_XP_003599606.1           KMEIVTGTGEVVNCSEEQNGELFYSVLGGLGQFGIITRARILLEPAPTMVKWIRVLYSDF    281
AtCKX1_AT2G41510.1              QLEIVTGKGEVVTCSEKRNSELFFSVLGGLGQFGIITRARISLEPAPHMVKWIRVLYSDF    276
CaCKX1_XP_004493454.1           QLEVVTGKGEVVTCSENRNADLFHGVLGGLGQFGIITRARISLESAPKRVKWIRVLYSDF    273
MtCKX1_XP_003625100.1           QLEIVTGKGEVVTCSENRNADLFHGVLGGLGQFGIITRARISLEPAPKMVKWIRVLYSDF    273
                                ::*:***.**::.*:: .* :** .************:*** ** **  ******** **

AtCKX6_AT3G63440.1              AAFAKDQEQLISAQGHKFDYIEGFVIINRTGLLNSWRLSFTAEEPLEASQFKFDGRTLYC    322
CaCKX6-like_XP_012574863.1      TAFTNDQERLISEE-NAFDYIEGFVIINRTGLLNNWRSSFNPQDPLQATHFKSDGKTLFC    323
MtCKX6_XP_003605865.1           TEFTRDQERLISEE-NAFDYIEGFVIINRTGLLNNWRSSFNPQDPVQASHFKSDGKTLFC    317
CaCKX6-like_XP_004514814.1      TAFTRDQEKLIFAE-KGFDYIEGFVIKNRTGLLNNWRSSFNPQDPVQASKFKSDGRTLYC    343
MtCKX6_XP_003599606.1           TAFTKDQEKLIFAE-KAFDYIEGFVIKNRTGLLNNWRLSFNPQDPVQASKFKSDGRTLFC    340
AtCKX1_AT2G41510.1              SAFSRDQEYLISKE-KTFDYVEGFVIINRTDLLNNWRSSFSPNDSTQASRFKSDGKTLYC    335
CaCKX1_XP_004493454.1           SLFTRDQEYLISLK-DTFDYIEGFVIINRTGILNSWRLSFDPKDPLQASQFNSDGKTFYC    332
MtCKX1_XP_003625100.1           SKFTRDQEYLISLK-DKIDYIEGFVIINRTGILNGWRLSFDPKDPLQASQFNSDGKTFYC    332
                                [illegible]

AtCKX6_AT3G63440.1              LELAKYLKQDNKDVINQEVKETLSELSYVTSTLFTTEVAYEAFLDRVHVSEVKLRSKGQW    382
CaCKX6-like_XP_012574863.1      LELAKYFNFQEMDIVNQEVERHLSHLNYIQSTLFQTEVTYVEFLDRVHVSEIKLRSKGLW    383
MtCKX6_XP_003605865.1           LELAKYFNFQQINIVNQDVERHLSRLNYIRSTLFQTEVTYVEFLDRVHVSEVKLRSKGLW    377
CaCKX6-like_XP_004514814.1      LELAKYFNHQETFAVNQDVEKHLSHLNYIPSTLFQTEITYVDFLDRVHISEVKLRSKGLW    403
MtCKX6_XP_003599606.1           LELAKYFNMEETLEVNQDIQKHLSHLNFIPSTLFQTEVTYVDFLDRVHISEVKLRSKGLW    400
AtCKX1_AT2G41510.1              LEVVKYFNPEEASSMDQETGKLLSELNYIPSTLFSSEVPYIEFLDRVHIAERKLRAKGLW    395
CaCKX1_XP_004493454.1           LEMAKYFNPDEVEVVNQGVDHLLSQLSYIPPTLFLSEVSYVEFLDRVHVSEKKLRAQGLW    392
MtCKX1_XP_003625100.1           LEMAKYFNPDEADVMNQDVDHLLSQLSYIPPTLFLSEVSYVEFLDRVHVSEKKLRAQGLW    392
                                **:.**:: ::   ::*   . **.*.::  *** :*: *  ******::* ***::* *

AtCKX6_AT3G63440.1              EVPHPWLNLLVPRSKINEFARGVFGNILTDTSNGPVIVYPVNKSKWDNQTSAVTPEEEVF    442
CaCKX6-like_XP_012574863.1      DVPHPWLNLFIPKSKIHSFAQLVFGNILTQTSNGPVLIYPVNKSKWDNRTSVVIPDEDIF    443
MtCKX6_XP_003605865.1           DVPHPWLNLFIPKSKIHSFAQFVFGNILTQTSNGPVLIYPVKKSKWDNRTSVVIPDEDIF    437
CaCKX6-like_XP_004514814.1      DVPHPWLNLLIPKSKIHNFAEVVFGHIVKETSNGPVLIYPVHKSKWDNRTSVAIPNEDIF    463
MtCKX6_XP_003599606.1           DVPHPWLNLFIPKSKIHNFAEVVFGNIVKETSNGPVLIYPVHKSKWDKRTSVVIPDEDIF    460
AtCKX1_AT2G41510.1              EVPHPWLNLLIPKSSIYQFATEVFNNILTSNNNGPILIYPVNQSKWKKHTSLITPNEDIF    455
CaCKX1_XP_004493454.1           EVHHPWLNLLIPRSEIHDFAEVVFGHILKDTSNGPILIYPVNQTRWNSKTSLVTPEEDVF    452
MtCKX1_XP_003625100.1           EVHHPWLNLLIPRSEIHDFAKEVFGNILKDTSNGPILIYPVNQTRWNSKTSFVTPEEDVF    452
                                :* ******::*:*.* .**  **.:*:....***:::***::::*..:**   *:*::*

AtCKX6_AT3G63440.1              YLVAILTSASPGSAGKDGVEEILRRNRRILEFSEEAGIGLKQYLPHYTTREEWRSHFGDK    502
CaCKX6-like_XP_012574863.1      YLVAFLSSADTSSNGSDSLEHILSQNKRILEVCERANLGVKQYLPQYNTQDEWRAHFGTH    503
MtCKX6_XP_003605865.1           YLVAFLTSAVPSSNGTDGLEHILSQNKRILEYCQRENLGVKQYLPHHNTQEEWRDHFGTK    497
CaCKX6-like_XP_004514814.1      YLVAFLASAVPSSNGCNGLEYILSQNKRILEYCERAHLGVKQYLPHYTTQEEWETHYGPK    523
MtCKX6_XP_003599606.1           YLVGFLAS----SSGPDELEHILSQNKRILEYCERAHLGVKQYLPHYTTQEEWQTHYGHK    516
AtCKX1_AT2G41510.1              YLVAFLPSAVPNSSGKNDLEYLLKQNQRVMNFCAAANLNVKQYLPHYETQKEWKSHFGKR    515
CaCKX1_XP_004493454.1           YLVAFLSSAVPFSTGENSLEYILNQNKRILDFCSHAQLNVKQYLPHYSTQEEWQAHFGSQ    512
MtCKX1_XP_003625100.1           YLVAFLTSAVPFSTGENSLEYILNQNKRILDFCTHAQLNVKQYLAHYDTQEEWQVHFGSQ    512
                                ***.:* *    * * : :* :* :*:*::: .    :.:**** :: *:.**. *:* :

AtCKX6_AT3G63440.1              WGEFVRRKSRYDPLAILAPGHRIFQKAVSYS-----------------------------    533
CaCKX6-like_XP_012574863.1      WEGFRQRKFVYDPFAILAPGQRIFQKTIIST-----------------------------    534
MtCKX6_XP_003605865.1           WEIFSQRKFVYDPFAILAPGQRIFQKTITTS-----------------------------    528
CaCKX6-like_XP_004514814.1      WQILLQRKSIYDPLAILAPGQGIFQKSITFS-----------------------------    554
MtCKX6_XP_003599606.1           WEIFKQRKSIYDPLAILAPGQGIFSKSITFS-----------------------------    547
AtCKX1_AT2G41510.1              WETFAQRKQAYDPLAILAPGQRIFQKTTGKLSPIQLAKSKATGSPQRYHYASILPKPRTV    575
CaCKX1_XP_004493454.1           WGAFVERKRAYDPLALLAPGHKIFQKAMSTFI----------------------------    544
MtCKX1_XP_003625100.1           WGAFVERKRTYDPLALLAPGHRIFQKAVSSSI----------------------------    544
                                *  : .**  ***:*:****: **.*:
```

(b)

(c)

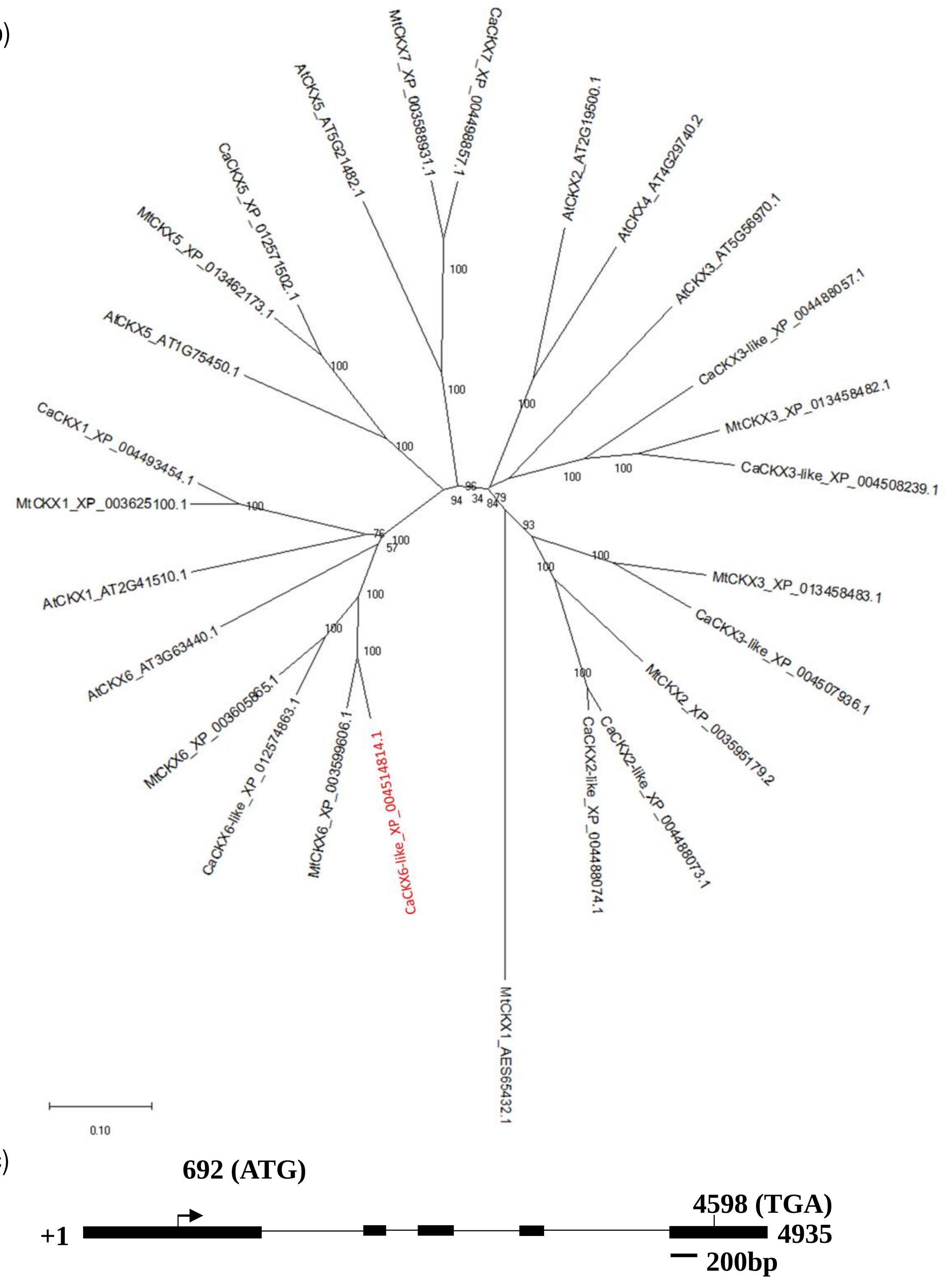


**Figure S1** Phylogenetic analysis of CKX proteins in *Arabidopsis*, *Medicago* and chickpea (a) ClustalW alignment of amino acid sequences of CKX1 and CKX6 orthologs in *Arabidopsis*, *Medicago* and chickpea. (b) Dendrogram displaying phylogenetic analysis of CKX proteins of *Arabidopsis*, Medicago and chickpea. Dendrogram is generated by maximum likelihood method as implemented in MEGA10.0.5 based on ClustalW alignment by protein sequences with 1000 bootstraps. The CaCKX protein (XP_00451481.1) used in this study is shown in red colour. (c) Schematic representation of gene structure of *CaCKX6* gene.

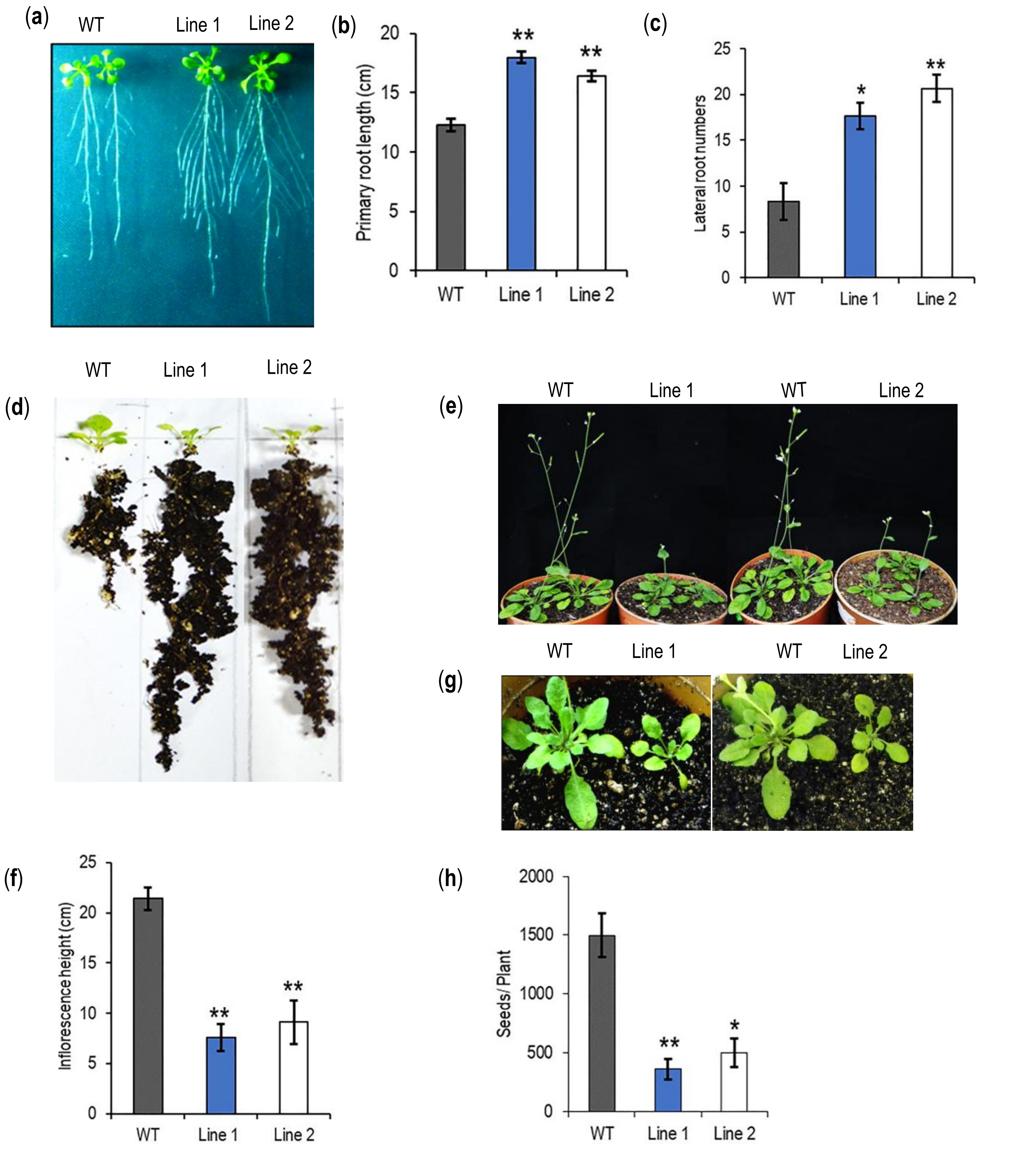


**Figure S2** Phenotypic effect of *CaCKX6* overexpression in *Arabidopsis*. (a-c) Phenotype, root length and lateral root number of 10 days post-germination (dpg)-old vertically grown *Arabidopsis* lines expressing *CaCKX6* under CaMV35S promoter. (d) Soil-holding capacity of 14 dpg-old pot-grown *35S::CKX6* (Line1 & Line2) plants. (e) Comparative phenotype of pot-grown 28 dpg-old WT (Col-0) and *35S::CKX6* (Line1 & Line2) plants. (f-g) Comparative phenotype and inflorescence height of pot-grown 42 dpg-old WT (Col-0) and *35S::CKX6* (Line1 & Line2) plants. (h) Comparison of seed numbers per plant of WT (Col-0) and *35S::CKX6* (Line1 & Line2) plants. Bars in represent means ± SE. Asterisks indicate statistically significant differences from WT determined using two-tailed Student's t-test (* for $p < 0.05$; ** for $p < 0.01$, *** for $p < 0.001$).

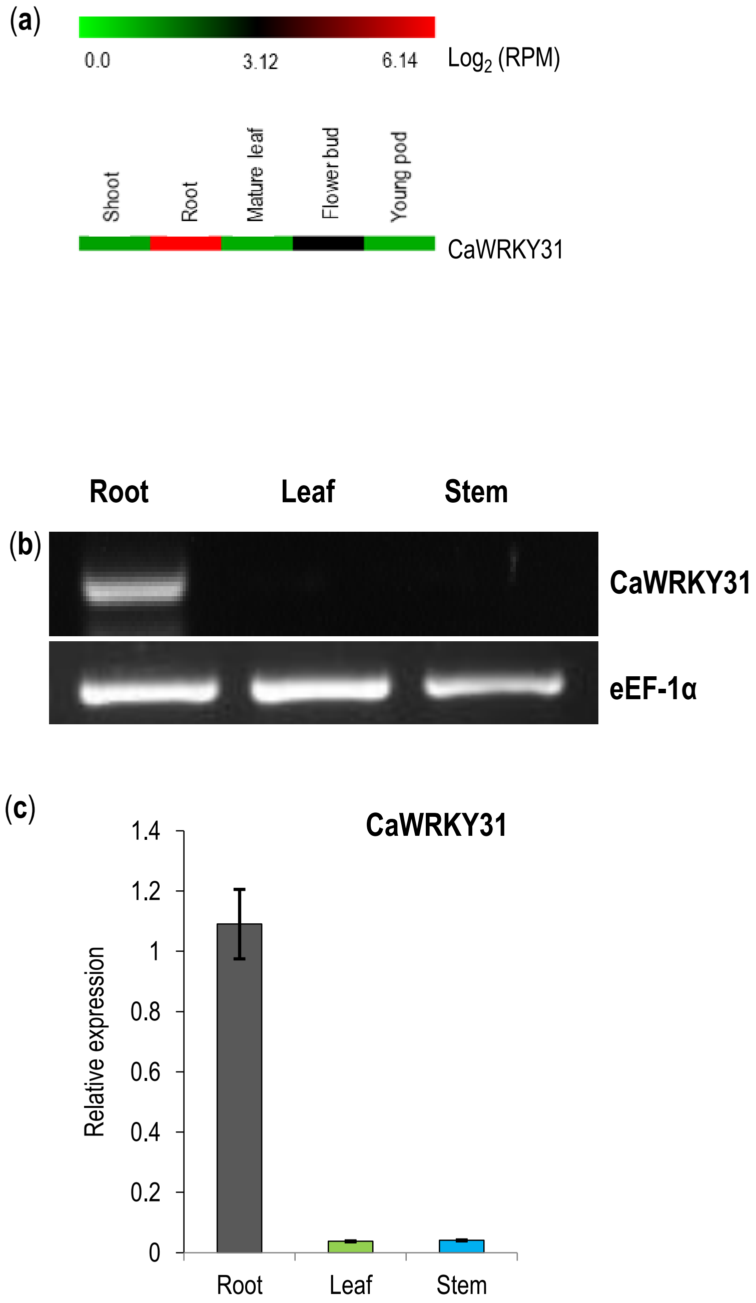


**Figure S3** Tissue-specific expression of *CaWRKY31* gene in chickpea. (a) Expression of *CaWRKY31* in various tissues as derived from transcriptome sequence (Garg et al. 2011). (b-c) Relative fold expression of *CaWRKY31* gene in various tissues of chickpea as determined by RT-PCR (32 cycles) and RT-qPCR.

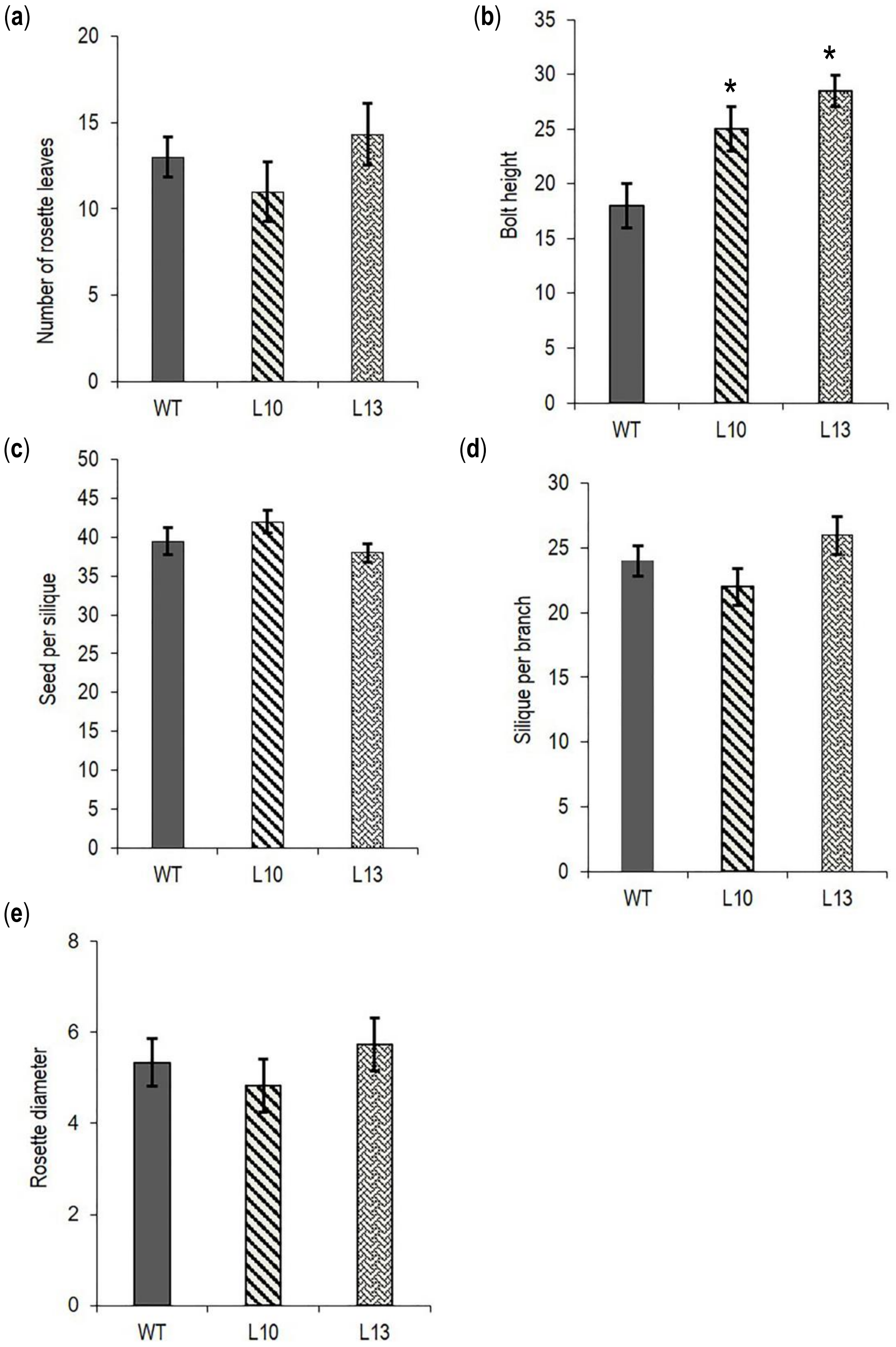


**Figure S4** Comparison of phenotypic parameters of WT and *W31::CKX6 Arabidopsis* plants. (a-e) Comparison of number of rosette leaves, bolt height, seeds per silique, silique per branch and rosette diameter, respectively, of 50 dpg-old pot-grown WT and transgenic *Arabidopsis* lines. Bars represent means ± SE. Asterisks indicate significant differences from the WT as determined by two-tailed Student's t-test (*for $p < 0.05$)

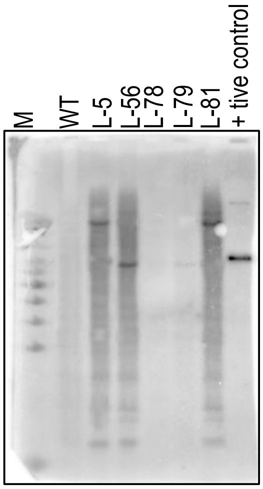


**Figure S5** Southern blot hybridization of the chickpea transgenic events. The figure shows genomic integration of *W31::CKX6* construct determined by Southern Blotting using a 196 base radiolabelled probe derived from the junction of *WRKY3*1 promoter and CDS of *CaCKX6*. DNA molecular weight marker (lane M), untransformed wild type chickpea plant (lane 1), positive control (vector construct) and different transgenic lines (lane 3-7) were shown.

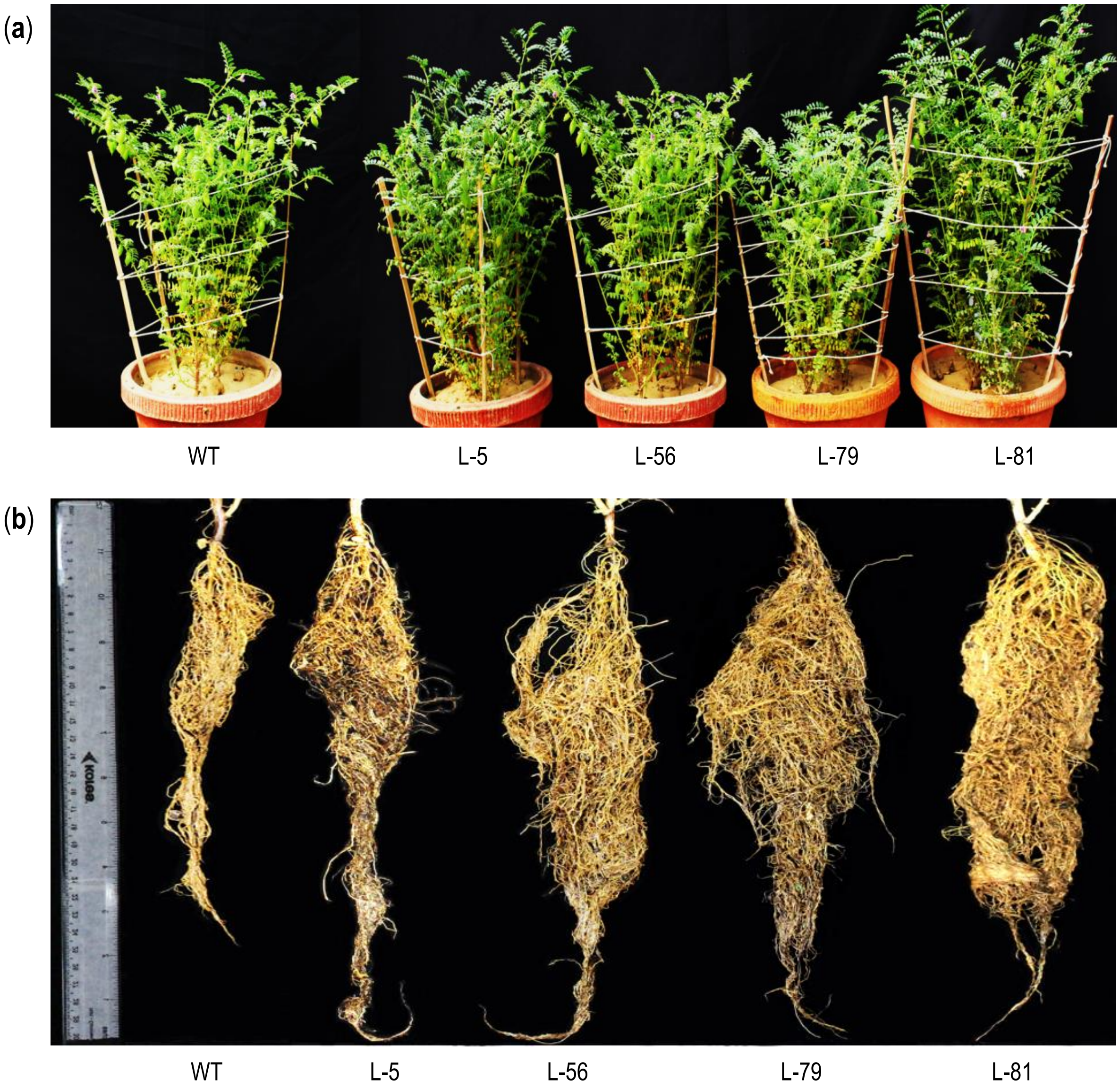


**Figure S6** Phenotypic observation of transgenic chickpea plants**.** (a-b) Shoot (120 dpg old) and root (155 dpg old) phenotype of pot-grown mature WT and transgenic lines.

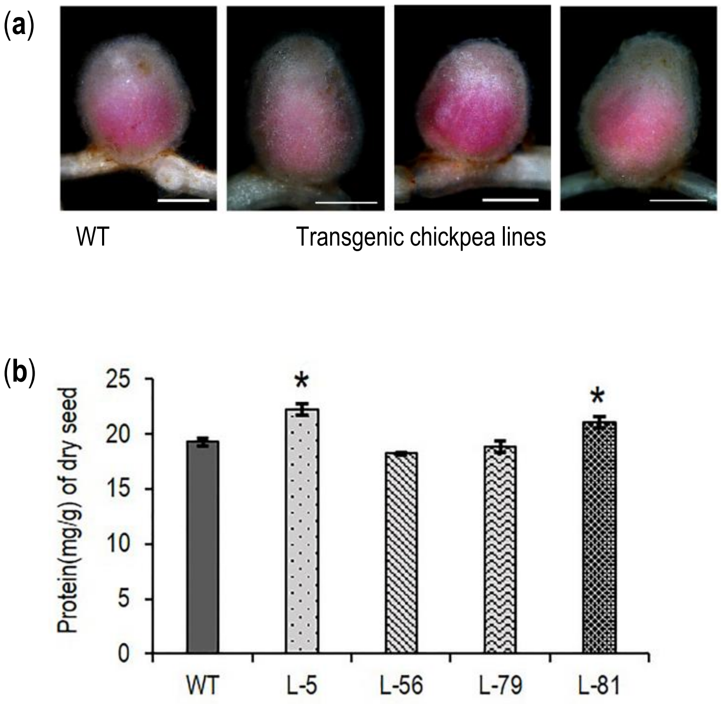


**Figure S7** Nodule phenotype and total seed protein estimation in transgenic plants. (a) Colored nodule formation in transgenic chickpea roots as an indicative of leghaemoglobin. (b) Total seed protein content in different chickpea plants. Bars represent means ± SE.

(a)

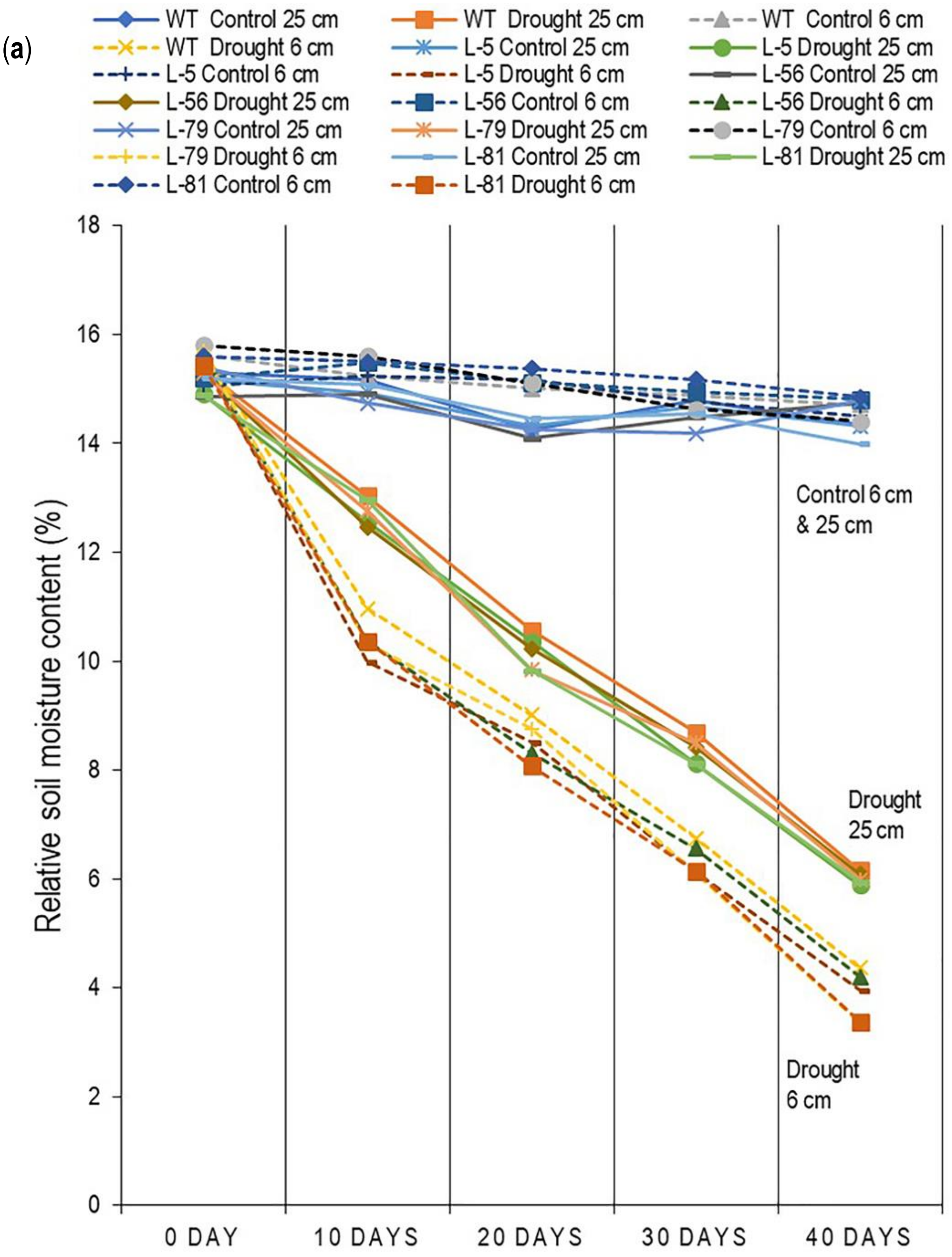


(b)

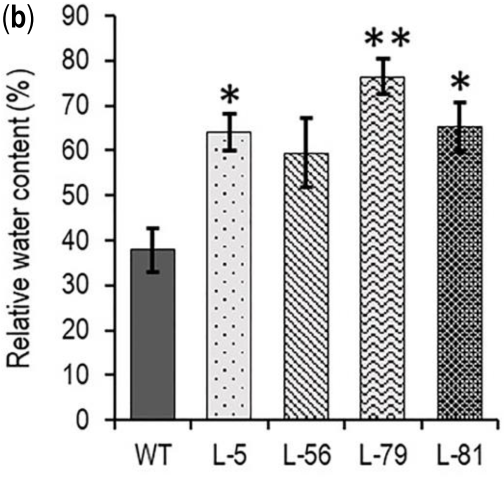


**Figure S8** Relative soil moisture content and leaf relative water content of control and drought-treated chickpea plants. (a) Measurement of soil moisture content by soil moisture meter at two different soil depth (6 cm and 25 cm) after withholding irrigation during drought treatment. (b) Relative water content (RWC) in third and fourth leaves from top of WT (PUSA 362) and transgenic chickpea lines at control and under drought-treated conditions. Bars represent means ± SE. Asterisks indicate significant differences from the WT as determined by two-tailed Student's t-test (*for $p < 0.05$; ** for $p < 0.01$).

## Supplementary table

### Table S1: Primers used in this study

| Primer name | Sequence (5' -3') |
|---|---|
| **CapWRKY31_gty_F** | CACCCTCCGTACTTATCCGGCTAT |
| **CapWRKY31_gty_R** | GAAAATCCAATGAGAGAGATTAAGACT |
| **CaCKX6_gty_F** | CACCATGCTTAACTTTAAGCTTCT |
| **CaCKX6_gty_R SCR** | ATGAGAAAGTAATTGATTTTTGAAA |
| **pCaWRKY31_F** | GCTCTAGACTCCGTACTTATCCGGCTA |
| **pCaWRKY31_R** | CCCGGGGAAAATCCAATGAGAGAGAT |
| **CaCKX6_F** | CCCGGGATGCTTAACTTTAAGCTTC |
| **CaCKX6_R** | GAGCTCTCATGAGAAAGTAATTGATTT |
| **CaEF1α_F** | TCCACCACTTGGTCGTTTTG |
| **CaEF1α_R** | CTTAATGACACCGACAGCAACAG |
| **M13_F** | CCCAGTCACGACGTTGTAAAACG |
| **M13_R** | AGCGGATAACAATTTCACACAGG |
| **GUS_F** | CTGTGGGCATTCAGTCTGGATC |
| **GUS_R** | GTTGGCAATACTCCACATCACC |
| **GUS Rev pBI121** | CACCAACGCTGATCAATTCCACAG |
| **CaMV35s_F** | CACTATCCTTCGCAAGACCCTCC |
| **AtACT2_F_RT** | TCAGATGCCCAGAAGTCTTGTTC |
| **AtACT2_R_RT** | GTGGATTCCAGCAGCTTCCA |
| **AtACT2_F** | GCCATCCAAGCTGTTCTCTC |
| **AtACT2_R** | GCTCGTAGTCAACAGCAACA |
| **NPTII_F** | ATGATTGAACAAGATGGATTGCACGC |
| **NPTII_R** | TCAGAAGAACTCGTCAAGAAGGCGA |
| **NCED2 RT_F** | TGCGTTAACGGCGTCTACCT |
| **NCED2 RT_ R** | TCCTGAAACTGGCTCGAACA |
| **CaABA-8'-OH RT_ F** | ATTTTTGCTGCTCGTGACACA |
| **CaABA-8'-OH RT_ R** | GGACACTAGGATTTCACCAAGGT |
| **CaP5CS1 RT_F** | GCTTCCTTGGCGATTCGA |
| **CaP5CS1 RT_F** | TAGCCTCCTTGCCTCCTTTCA |
| **CaCKX6 RT_F** | AACTTTAAGCTTCTTCACC |
| **CaCKX6 RT_R** | AACTGATTTTGGATGAAGTACTG |

# Appendix:

## Appendix S1 (1500 bp CaWRKY31 Promoter)

CTCCGTACTTATCCGGCTATAAGTATGTCCAAGTCAAAGTTACAAGAATGTAGGGACCCAC
ACTTAGTCGTAACTATGTAGGACCCACTTGGCCCATCAGAACCATTGACTTTTGACTATATT
TTTCACGGACCAATAGGGGCGACGAATTTTGAGAACGCGTCAATCTCCTTCCTTCGTGCTT
TTCTTCATCCACAATAAATAAACACTCATTTTTTTAATAATTAAAATCATCACTTCAATTTTTT
TCAAATAAATTAGATCAACACCTCATCAATTTGTAAAACGTGTAAATCAATCATAGTATGTAT
AAACTATAAACAATAACAACCACGAAAACAAAGTTTGTTGACTCCCAAATTCtCTTTAGAA
ATTCAAAATTTATAGTATGTGAGTATATTTAtATTTTTTTATATACTTTTTTTATTTAAAAATAACA
ATGTGTTTTCTTCTTGATGGGTGAAAATGGTTGTGTATGAACTAGTCAACATAAAAATGTTT
TGGACATGACTGAAAAATGTTTTGTACATTACTTATTAATGTTGGTAAATGACTTTCTTAAAA
TCAAAAGTACTCAAATTAAGGATGAAAAATAGATTAAAAATAAAATAATTTTTTAGCTATTGA
TGAAAAAAGTGAATACTATTGGTAGAATCCCATAAATATAAAAAAAATATTACCGAATGATTG
TATATTTGATTGCAATGTACCATATCCAACAAAACTATAACAAAACAACCACATAACTTTTAG
ATTCTAAAAATAATGTTATAATTATTTTTGAACCTATATTTAAGATAAATTCTTTTTTCTTTTGA
CATAATTTATGATAATTCTATTACAAAAAAAAAAGTTAAAAAaTATAGGTTAGTCTTTTTTATAtA
TTTtATCCTAAAACATATTAAACATGTTTTTTtAAGTATGAAATTGATCTAAATTTCATAAATAA
AATCATGTAAATAAATAATATATTTAAATTAAATAAATAAATTTTGGATGAAATAACATGAATT
TCTATTAACTTAATTATAGCACATACTCGAATTTAATAGTAAAATATAATAATATTATAAAAAA
GTTAAAAAGTTTTATTGTCCATATCCGACTCAACACAAGCTATTAGTCTTCACAAGGATGCA
ATATATTTACTTTACAAAAAGTTTAGACAAATAAAAAAATATAAAAGTATTATTTATTTAATAA
AAAATTGTTTGGTGTTAGAATGGATCCATGACACATTTATTACAGGCATAGACTATATGACTT
TTGTGAATTATTTTCCATGTATTCTCTTGTATTATGTCAAGAAAAAAACTGAAATGTAATACC
ACAACAACATTAATGCAAAAGAAAGAAATAAAAACACATTTTTATTTTCACAACTAAAAACA
ATTTTCTCATCAAGTCACAAACTTCCTCCATTTTATTTGCCGATTCAATTGCATCAATTCTAA
CACTTCAAATACTTCTCTCTAGTCTTAATCTCTCTCATTGGATTTTC

## Appendix S2 (196 bp probe for Southern blotting)

CTCATCAAGTCACAAACTTCCTCCATTTTATTTGCCGATTCAATTGCATCAATTCTAACACT
TCAAATACTTCTCTCTAGTCTTAATCTCTCTCATTGGATTTTCCCCGGGATGCTTAACTTTA
AGCTTCTTCACCCTTTAACTACCAACAACATATCTTCCATAAAAACATTTAGATATCCATCA
TTTAGCCTTG

## Appendix S3

### Histochemical GUS staining and protein localization

Histochemical GUS staining assay was performed as previously described before (Jefferson *et al.*, 1987). In brief, roots and shoots of chickpea seedlings were vacuum infiltrated with GUS staining solution (50 mM sodium phosphate buffer pH 7.0, 2 mM EDTA, 0.12 % Triton-X, 0.4 mM ferrocyanide, 0.4 mM ferricyanide, 1.0 mM 5-bromo-4-chloro-3-indoxyl-beta-D-glucuronide cyclohexyl ammonium salt (X-Gluc) (Sigma-Aldrich, St. Luis, MO) for 15 min and incubated in dark at 37$^{o}$C. Tissue was cleared by incubating with a saturated solution of chloral-hydrate at 65°C for 1-2 h and analyzed by light microscopy. For subcellular localization of *CaCKX6*, a previously published method (Meena *et al.*, 2019) was followed. The organelle marker for the endoplasmic reticulum (CD3-959 mCherry) was used (Nelson *et al.*, 2007).

### RNA isolation, RT-qPCR and CKX activity assay

Total RNA was extracted from tissues using TRIzol reagent (Invitrogen) following the manufacturer's protocol. Gene-specific primers were used to detect transcripts by RT–qPCR. All primers were designed using PRIMER EXPRESS version 3.0 (Applied Biosystems, Foster City, CA) with default parameters. All the primers used in this study are listed in Supplementary Table S1. Quantitative real-time PCR (RT-qPCR) was carried out according to the procedure described before (Meena *et al.*, 2015). *Arabidopsis ACTIN2* (At3g18780) and chickpea *EF-1α* (NM_001365163.1) was used as reference genes for RT-qPCR. The CKX enzyme activity assay was performed by using root material from three-week -old soil-grown transgenic chickpea plants according to Caros *et al*., (1995). Enzyme activity was calculated as μM of 3-methyl-2-butenal per mg of protein extract. The protein contents (Bradford, 1976) and the enzyme activity were carried out according to the procedure described before (Ramireddy *et al.*, 2018a).

### Phytohormone assay

The endogenous level of plant hormones was estimated by LC-MS using a modified protocol. Roots were crushed in liquid $N_2$ and lyophilized. Approximately 20-30 mg of powder was suspended in extraction solvent (2-propanol/Milli Q water/concentrated HCl; 2:1:0.002/v/v/v) with shaking at 500 rpm for 30 min at 4°C. Thereafter, 1 ml of chilled dichloromethane was added with continued shaking for another 30 minutes. The organic and aqueous phase was separated by centrifugation at 13000*g* for 5 min at 4°C. The aqueous phase was separated in a fresh tube and dried *in vacuo*. These samples were further dissolved in chilled methanol followed by centrifugation at 13000*g* for 5 min. Thereafter, samples were transferred for analysis using Exactive$^{Tm}$ plus Orbitrap mass spectrometer (Thermo Fisher, USA) coupled with UPLC (Waters, Milford, MA USA). Details of CK extraction, separation, and quantification including external and internal standards have been described before (Kumari *et al.*, 2017). The detection of ABA and t-Zeatin were performed using electrospray ionization (ESI) in negative ion mode and Turbo Ion spray source in positive ion mode, respectively. The levels of endogenous hormones were quantified by using standard curves for the hormones, ABA & trans-zeatin.

**Supplementary references**

Bradford, MM., (1976). A rapid and sensitive method for the quantitation of microgram quantities of protein utilizing the principle of protein-dye binding. *Analytical Biochemistry*, **72**, 248-254.

Caros, A., Libreros-Monotta, & Peter A. Tipton. (1995). A colorimetric assay for cytokinin oxidase. Analytical Biochemistry, 231, 339-341.

Jefferson, RA., Kavanagh, TA., & Bevan, MW. (1987). GUS fusions: beta-glucuronidase as a sensitive and versatile gene fusion marker in higher plants. EMBO Journal, 20, 3901-3907.

Kumari, A., Kamalika, R., Sadhna, S., Pandey, AK., Sreelakshmi, Y., & Sharma, R. (2017). Metabolomic homeostasis shifts after callus formation and shoot regeneration in tomato. PLOS ONE, 12, e0176978.

Meena, MK., Ghawana, S., Sardar, A., Dwivedi, V., Khandal, H., … Chattopadhyay, D. (2015). Investigation of genes encoding Calcineurin B-like protein family in legumes and their expression analyses in chickpea (Cicer arietinum L.) PLoS ONE, 10, e0123640.

Meena, MK., Vishwakarma, NK., Tripathi, V., & Chattopadhyay, D. (2019). CBL-interacting protein kinase 25 contributes to root meristem development. Journal of Experimental Botany, 70, 133-147.

Nelson, BK., Cai, X., & Nebenführ, A. (2007). A multicolored set of in vivo organelle markers for co-localization studies in *Arabidopsis* and other plants. *The Plant Journal*, **51**, 1126–1136.

Ramireddy, E., Hosseini, SA., Eggert, K., Gillandt, S., Gnad, H., von, N., & Schmülling, T. (2018a). Root engineering in barley: Increasing cytokinin degradation produces a larger root system, mineral enrichment in the shoot and improved drought tolerance. *Plant Physiology*, **177**, 1078-1095.
